# Supplementary material for: Surface wetting is a key determinant of α-synuclein condensate maturation
Source: Commun Chem. 2025 Nov 27;8:379. doi: 10.1038/s42004-025-01764-z (PMC12661020; doi:10.1038/s42004-025-01764-z)
Supplement: Supplementary file 2 — Supplementary Information [file 42004_2025_1764_MOESM2_ESM.pdf]

Supplementary Information for

Surface Wetting Is a Key Determinant of α-Synuclein Condensate Maturation

Rebecca J. Thrush,[1,2] Devkee M. Vadukul,[1] Siân C. Allerton,[1,2] Marko Storch,[3,4] and Francesco A. Aprile[1,2]*

[1]Department of Chemistry, Molecular Sciences Research Hub, Imperial College London, London, UK.
[2]Institute of Chemical Biology, Molecular Sciences Research Hub, Imperial College London, London, UK.
[3]London Biofoundry, Translation and Innovation Hub, Imperial College London, London, UK.
[4]Department of Infectious Disease, South Kensington, Imperial College London, London, UK.

*To whom correspondence should be addressed: f.aprile@imperial.ac.uk +44(0)20 7594 5545

This PDF file includes:

Supplementary Methods
Supplementary Tables 1 and 2
Supplementary Figures 1 to 37
Supplementary References

Other Supplementary Information for this manuscript includes:

Supplementary Movies 1 to 4

Supplementary Methods

Protein expression and purification

FL α-syn (residues 1-140) was expressed and purified as detailed previously.[1] Briefly, pT7-7 α-syn FL plasmid (a gift from Hilal Lashuel, Addgene, USA[2]) was transformed into BL21-Gold (DE3) competent *Escherichia coli* (*E. coli*) cells (Agilent Technologies) according to the manufacturer's instructions and previous methodology. Transformed cells were grown (37 °C, 200 rpm shaking) in LB media containing ampicillin (100 µg/ml) to an OD600 of ~ 0.7 before FL α-syn expression induced with 1 mM IPTG overnight (28 °C, 200 rpm shaking). The cells were then harvested by centrifugation and resuspended in buffer A (20 mM Tris-HCl, 1 mM EDTA, pH 8.0) including a protease inhibitor tablet (EDTA-free). Cells were lysed by sonication on ice before cell debris was removed by centrifuged (18,000 rpm, 45 min). The lysate was then boiled at 80 °C for 20 min, followed by another round of centrifugation (18,000 rpm, 30 min). Next, streptomycin sulphate (10 mg/ml) was gradually introduced to the supernatant. The mixture was incubated with rotation before further centrifugation (18,000 rpm, 30 min). FL α-syn was precipitated by the slow addition of ammonium sulphate (360 mg/ml) on ice, followed by incubation with rotation, and a final centrifugation (18,000 rpm, 30 min). The pellet was collected, re-suspended and dialyzed into buffer A overnight. The sample was then loaded onto an anion exchange chromatography column (HiPrep Q HP 16/10, Cytiva, USA) and further purified using a gradient elution with buffer B (20 mM Tris-HCl, 1 M NaCl, 1 mM EDTA, pH 8.0). The FL α-syn protein fractions, as determined by sodium dodecyl sulphate–polyacrylamide gel electrophoresis (SDS-PAGE), were then dialyzed into phosphate buffered saline (PBS, pH 7.4) overnight, before being passed through a gel filtration column (HiLoad 26/600 Superdex 75 pg, Cytiva, USA). The pure fractions, determined by SDS-PAGE, were combined and the concentration determined by Ultraviolet–visible (UV-Vis) spectroscopy absorbance at 275 nm (ε_{275nm} = 5600 $M^{-1}cm^{-1}$).

AcFL α-syn expression and purification was performed as described above, except that the pT7-7 α-syn FL plasmid was co-transformed with the pNatB plasmid, and the resulting cells were grown in LB medium supplemented with ampicillin and chloramphenicol (100 µg/ml each).

To generate 11-140, 14-140 and 19-140 α-syn, deletion polymerase chain reaction (PCR) was performed on the pT7-7 Int7-140αSyn plasmid, which encodes a protein sequence where an intein is fused to the N-terminus of residues 7-140 of α-syn,[1] eliminating the need for a non-native starting Met in the protein sequences. PCR was carried out using the Q5 high-fidelity DNA polymerase, the 5' phosphorylated primer pairs and their corresponding annealing temperatures (T_a), listed in Supplementary Table 2. The template DNA was then digested with DpnI. Following sample purification using a NucleoSpin Gel and PCR Clean-up kit (MACHEREY-NAGEL, Germany), T4 DNA ligase was used to ligate the 5' phosphorylated blunt-end DNA fragments. All steps were carried out according to the relevant manufacturer's instructions. The resultant plasmids were then transformed into XL10-Gold Ultracompetent E. coli cells and purified using a QIAprep Spin Miniprep Kit. Mutagenesis success was determined by sequencing (GENEWIZ). Following several failed attempts to generate the 11-140 α-syn plasmid, Genscript performed the mutagenesis on our behalf.

Both variant plasmids were then transformed into BL21-Gold (DE3) cells, we found that increasing initial incubation on ice from 30 to 90 min increased transformation efficiency. The intein-α-syn fusion proteins corresponding to 11-140 and 19-140 α-syn were then expressed and the cell lysate harvested as described for FL α-syn. Subsequently, the fusion proteins were immobilized on a chitin resin before the target α-syn variant was cleaved, eluted and further purified by size-exclusion chromatography as previously described.[1]

The N-terminal residue of 5-140 α-syn is Met and so the variant does not require intein conjugated expression. To generate the mutated plasmid the above deletion PCR process was applied to the pT7-7 α-syn FL plasmid using the 5' phosphorylated primer pair listed in Supplementary Table 2. 5-140 α-syn was then expressed and purified as detailed for the FL protein.

Fluorescent labelling

FL α-syn was covalently tagged at its lysine residues using NHS-Rhodamine (ex. 552 nm, em. 575 nm). The probe was added to 100 µM α-syn in ~ 8-fold molar excess, and the reaction was allowed to proceed at RT (~ 4 h, gentle mixing). Subsequently, excess dye was removed by extensive dialysis into PBS, pH 7.4 (overnight, 4 °C). For subsequent assays, it was sufficient to assume no significant change in α-syn concentration had occurred during this process.

Electrospray ionization mass spectrometry

Purified protein samples (~ 50 µM, diH2O) were analyzed by electrospray ionization mass spectrometry (ESI-MS). ESI-MS was performed at the Chemistry Mass Spectrometry Facility, Molecular Sciences Research Hub, Department of Chemistry, Imperial College London. The spectra were plotted using GraphPad Prism version 10.0.3 (GraphPad Software).

Transmission election microscopy

A sample of the washed insoluble fraction taken at the endpoint of a dispersed solution aggregation assay, or of the whole solution taken at the endpoint of an aggregation assay under phase separation conditions, was applied to carbon films on 300 mesh copper grids (Agar Scientific Ltd., UK). The grids were quickly washed with diH2O, negatively stained with 2 % (w/v) uranyl acetate and washed again. Sample application, washing and staining steps were performed in rapid succession to ensure that the samples did not dry out between steps as dehydration has been shown to destabilize amyloid fibrils, leading to changes in their appearance if rehydrated.[3,4]

The grids were imaged using the Tecnai 12 Spirit transmission electron microscope (Thermo Fisher Scientific (formerly FEI), USA) available at the Electron Microscopy Centre, Centre of Structural Biology, Imperial College London. Where specified fibril lengths and widths were measured using the straight-line selection drawing tool in Fiji, with the data plotted using GraphPad Prism version 10.0.3. Only distinct amyloid fibrils that were clearly distinguishable as individual structures, not fibril bundles or clusters, that were wholly within the image were measured and a similar number of images at similar magnifications were quantified across variants. Because fibrils were identified by the presence of stain along their edges and given the limited lateral resolution of negative-stain TEM, width measurements were associated with a higher relative error than length measurements. To account for this, we analyzed ≥292 fibrils per condition, with statistical significance assessed using ANOVA.

Dot-blot analysis

The presence of α-syn in the insoluble aggregates formed following aggregation under phase separation conditions was determined by dot-blot analysis. The soluble and insoluble protein fractions at the endpoint of the aggregation assay were separated by centrifugation (30 min, 14,000 rpm). The insoluble pellet was resuspended in a minimum volume of PBS before 5 replicates of 3 µl aliquots were transferred to a nitrocellulose membrane (0.45 µm) and allowed to dry. The membrane was then blocked (1 h, 4 °C) with a solution of milk powder (5 %) dissolved in PBS-T (PBS supplemented with 0.1 % Tween 20). After washing with PBS-T, the membrane was incubated (overnight, 4 °C) with the anti-α-syn primary antibody MJFR1 (diluted 1:1000 in PBS-Tween, Abcam, UK). Following rigorous washing with PBS-T, the membrane was incubated (1 h, RT) with the goat anti-rabbit IgG (H + L) highly cross adsorbed secondary antibody, conjugated to the fluorophore Alexa Fluor plus 555 (diluted 1:5000 in PBS-Tween, Thermo Fisher Scientific, USA). Finally, the membrane was washed with PBS-T and imaged with a Typhoon FLA 9000 (Cytiva, USA). As a control, the same dot-blot protocol was used to analyze the reactivity of the primary antibody against 25 µM PLK in PBS.

Circular dichroism

The far-UV circular dichroism (CD) spectra of fresh monomeric protein (20 µM) or the insoluble aggregate pellet (~ 10 µM) taken from the endpoint of a ThT aggregation assay under phase separation conditions were recorded. The insoluble fractions were obtained by centrifugation, and their concentration estimated using UV-Vis spectroscopy absorbance at 275 nm (ε_{275nm} = 5600 $M^{-1}cm^{-1}$) following denaturation in 4 M guanidinium chloride for 2 h at RT. Spectra of all samples were taken using a Chirascan v100 (Applied Photophysics Ltd, UK) from 200 to 250 nm with a 0.5 nm step, 1 nm bandwidth, 1 s per time point and 5 accumulations. A PBS background spectrum was subtracted from each sample spectrum, with data extracted from the same assay background corrected using the same buffer spectrum. For monomeric protein samples the raw data (in mdeg) were converted to mean residue ellipticity (MRE, units deg cm^2 $dmol^{-1}$) using:[5]

$$\text{MRE} = \text{mdeg} \div (l \times c \times (n-1)) \qquad \text{equation (1)}$$

where mdeg is the raw data (in mdeg), *l* is the cuvette pathlength (in mm), *c* is the sample concentration (in M), and *n* is the number of amino acids. The data were plotted using GraphPad Prism version 10.0.3.

Dispersed solution aggregation assay

To monitor all three phases in the amyloid aggregation, monomeric protein was aggregated and monitored by fluorescence spectroscopy using a previously described protocol.[1] Briefly, monomer solutions (50 µM) were aggregated with ThT (20 µM) and NaN_3 (0.02 %) in PBS (pH 7.4). 170 µl of sample (in triplicate) was loaded into a 96 well full-area µClear plate (non-binding, clear bottomed, #655906, Greiner Bio-One, Austria), sealed with an aluminum film and incubated at 37 °C for ~ 70 - 140 h in a CLARIOstar Plus microplate reader (BMG Labtech, Germany). Aggregation was promoted by linear shaking (300 rpm, 300 s before each cycle) and addition of a single borosilicate bead (3 mm diameter) per well. Fluorescent intensity measurements were taken every 520 s using spiral averaging (5 mm diameter) and excitation 440 nm, dichroic 460 nm, and emission 480 nm filters, 3 gains and 50 flashes per well. All sample spectra were background corrected by subtracting the spectrum of buffer alone (i.e., 20 µM ThT and 0.02 % NaN_3 in PBS), with data extracted from the same assay background corrected using the same buffer spectrum.

The data were plotted using GraphPad Prism version 10.0.3. The individual biological repeats were fit with sigmoidal, 4PL, X is concentration standard curves provided by the software. t_{50} was estimated as the time (x-value) at which 50 % maximum fluorescence intensity was reached. t_{lag} was estimated by extrapolation of the tangent at t_{50}. The steepness of the curve was estimated by the Hillslope value, where Prism assigns a standard sigmoidal receptor binding curve a Hillslope value of 1.0, while a steeper curve has a higher Hillslope value, and a shallower curve has a lower Hillslope value.

To estimate ɣ for FL and 5-140 α-syn, this assay was repeated with 10, 25, 50, 75 and 100 µM protein. The data were uploaded to AmyloFit 2.0 where the log of initial monomer concentration (m0) was calculated alongside the log of the half-life of aggregation (t_{50}).[6] All data were plotted using GraphPad Prism. The double logarithmic plot was fitted with a simple linear regression and ɣ estimated as the gradient. As ɣ is not constant for 5-140 α-syn, the data was fit with two straight lines corresponding to two different ɣ values.

Lipid-induced aggregation assay

Lipid vesicles were produced based on a previously described protocol.[7] Briefly, 1,2-dimyristoyl-sn-glycero-3-phospho-L-serine (sodium salt) was dissolved in 20 mM phosphate buffer (PB), pH 6.5 at ~ 70 °C for 2 h. The lipids were then freeze-thawed using liquid nitrogen and a ~ 50 °C water bath 5 times before being extruded 11 times through a membrane (100 nm pore diameter) at ~ 70 °C. Subsequently, monomer solutions (50 µM) were aggregated in PB (pH 6.5) in the presence of DMPS lipid vesicles (250 µM), ThT (20 µM) and NaN_3 (0.02 %). 170 µl of each sample (3 replicates) was loaded into a 96 well full-area µClear plate, sealed with aluminum foil, and incubated at 37 °C for ~ 40 h in a FLUOstar Omega microplate reader (BMG Labtech, Germany). Fluorescent intensity measurements were taken using spiral averaging (3 mm diameter), excitation 440-10 nm, dichroic 460 nm and emission 480-10 nm filters, 4 gains and 50 flashes per well. All sample spectra were background corrected by subtracting the spectrum of buffer alone (i.e., 20 µM ThT and 0.02 % NaN_3 in PB), with data extracted from the same assay background corrected using the same buffer spectrum. The size homogeneity of the lipid vesicles was determined by dynamic light scattering (DLS). All data were plotted using GraphPad Prism version 10.0.3.

Seeded aggregation assay

Pre-formed fibril seeds were generated either under phase separation conditions (*i.e.*, 60 µM monomeric FL or truncated α-syn with 25 µM PLK in phase separation buffer) or by shaking (~ 600 rpm) monomeric FL α-syn (~ 200 µM with NaN_3 (0.02 %) in PBS, pH 7.4) at 37 °C for ~ 5–10 days. The soluble and insoluble protein fractions were then separated by centrifugation (30 min, 14,000 rpm), the soluble fraction removed, and the pellet resuspended in fresh PBS, pH 7.4. Centrifugation and pellet resuspension were repeated before the final protein concentration was estimated using UV-Vis spectroscopy absorbance at 275 nm (ε_{275nm} = 5600 $M^{-1}cm^{-1}$) after denaturation in 4 M guanidinium chloride for 2 h at RT.

Seeded aggregation assays were carried out based on previously described protocols.[8-10] When performing the fibril elongation assays, the relevant pre-formed fibril solution(s) were diluted to 50 µM in PBS, pH 7.4 and probe sonicated on ice at 20 % power, 15 s pulse, 15 s rest for 4 cycles. Monomeric protein (50 µM) was then incubated with these short pre-formed α-syn fibrils (5 µM), ThT (20 µM) and NaN_3 (0.02 %) in PBS, pH 7.4. Monomeric α-syn was either incubated with fibrils composed of the corresponding variant (formed via phase separation) or with fibrils composed of FL α-syn (formed via shaking).

To perform secondary nucleation experiments, the FL α-syn pre-formed fibril stock formed via shaking was diluted to 5 µM in 20 mM acetic acid (NaCl 150 mM, pH 4.8) and probe sonicated on ice at 20 % power, 5 s pulse, 5 s rest, 3 cycles. Monomeric protein (50 µM) was also buffer exchanged into 20 mM acetic acid (NaCl 150 mM, pH 4.8), and then incubated with these long pre-formed FL α-syn fibrils (50 nM), ThT (20 µM) and NaN_3 (0.02 %).

For elongation assays using fibrils formed via phase separation, 100 µl of sample (3 replicates) was loaded into a 96 well full-area glass bottom plate, sealed with aluminum foil and incubated for ~ 65 h at 37 °C in a CLARIOstar Plus microplate reader under quiescent conditions. For elongation and secondary nucleation assays using FL α-syn fibrils formed via shaking, 170 µl of sample (3 replicates) was loaded into a 96 well full-area µClear plate, sealed with aluminum foil and incubated for ~ 40 h at 37 °C in a FLUOstar Omega microplate reader under quiescent conditions. Fluorescent intensity measurements were taken using the settings described above for the lipid-induced aggregation assay. All fibril elongation or secondary nucleation sample spectra were background corrected by subtracting the spectrum of buffer alone (i.e., 20 µM ThT and 0.02 % NaN_3 in either PBS or 20 mM acetic acid, respectively), with data extracted from the same assay background corrected using the same buffer spectrum. The size homogeneity of any sonicated pre-formed fibril seeds was determined by DLS. All data were plotted using GraphPad Prism version 10.0.3.

Monomer conversion analysis

The soluble and insoluble protein fractions before and after a dispersed solution aggregation assay were separated by centrifugation (30 min, 16,900 x g). The soluble fraction was then extracted and analyzed by SDS-PAGE. For each sample, the mean grey value of the band corresponding to the relevant monomeric protein variant was measured using Fiji.[11] Percentage conversion of monomer into

insoluble aggregates was estimated by comparison of the band intensity before and after aggregation. The data were plotted using GraphPad Prism version 10.0.3.

Condensate volume and concentration analysis

To estimate the volume fraction of condensates formed by each variant, the total condensate area per image, after 15 mins incubation, was divided by the total image area to obtain a % area value. To account for potential variations in object identification due to differences in contrast between images a mean % area value over the 3 z-stack images taken per replicate was calculated, with three biological replicates analyzed per variant.

The centrifuge parameters required to separate the dense-phase, *i.e.*, the condensates, from the dilute-phase was estimated using a previously published equation:[12]

$$n.g = 6\pi\eta r.h \div t.(p_d\text{-}p_s)V \qquad \text{equation (2)}$$

where n.g is the centrifugal RCF value, η the solvent viscosity, r the condensate radius, h the distance the condensates sediment, t the time, p_d the density of protein inside the condensate, p_s the solvent density and V the condensate volume. We approximated that the density of condensates is ~1.1 g cm^{-3} (at 37°C) and the density of 10 % PEG-8000 is ~1.01 g cm^{-3} (at 37°C).[13,14]

To sediment the smallest condensates with a radius of 0.28 µm by 1.5 cm in a 10 % PEG-8000 solution with a viscosity of ~7.5 mPa.s using an RCF value of 3000 xg would take 6.6 h.[13]

Thus, 200 µl of monomeric α-syn (0 or 60 µM) with PLK (0 or 25 µM) was equilibrated in phase separation buffer for 15 mins before the samples were centrifuged at 3000 xg for 7.5 h. Following sedimentation, 150 µl of supernatant, *i.e.*, the dilute-phase, was removed and its absorbance measured from 600-200 nm by UV-Vis spectroscopy. The spectra were buffer subtracted and then baseline corrected at 300 nm before the dilute-phase concentration was determined using the absorbance at 275 nm (ε_{275nm} = 5600 M^{-1}cm^{-1}).

The concentration of α-syn in the dense-phase was estimated using the total condensate volume and the dilute-phase concentration. The data were plotted using GraphPad Prism version 10.0.3.

Statistical analysis

Statistical significance was performed on the $t_{lag,}$ Hillslope and fibril length and width values extracted from the dispersed solution aggregation assay kinetics and TEM. A Welch and Brown-Forsythe ANOVA with multiple comparisons against the control (FL α-syn) was used where $^{ns}p \geq 0.05$, $0.01 \leq {}^{*}p < 0.05$, $0.001 \leq {}^{**}p < 0.01$, $0.0001 \leq {}^{***}p < 0.001$ and $^{****}p < 0.0001$.

Statistical significance was also performed on the half-life (t_{50}) of the decay in object count values, the condensate volume and dilute- and dense-phase concentration data acquired under phase separation conditions. A Welch and Brown-Forsythe ANOVA with multiple comparisons against the control (FL α-syn) was used with the same p values listed above.

Supplementary Table 1.

Schematic representation of the N-terminally modified α-syn variants selected for analysis[1].

α-Syn variant	ΔCharge	ΔHydrophobicity*	N-terminus
FL (1-140)	0	0	H_3N-MDVFMKGLSKAKEGVVAAAE
AcFL	-1	-	CH_3CO–MDVFMKGLSKAKEGVVAAAE
5-140	+1	- 5.4	H_3N-MKGLSKAKEGVVAAAE
11-140	-1	- 2.1	H_3N-AKEGVVAAAE
14-140	-1	+ 3.5	H_3N-GVVAAAE
19-140	-1	- 8.1	H_3N-AE

1. The first 20 residues of FL α-syn are shown with neutral residues in black, positive residues in green, negative residues in red. Subsequently, N-terminally acetylated α-syn and N-terminally truncated variants 5-140, 11-140, 14-140 and 19-140 α-syn are shown with their corresponding sequences. Resultant change in charge (ΔCharge) and in hydrophobicity (ΔHydrophobicity) of each variant, relative to the FL protein, is shown. *Calculated according to the Kyte-Doolittle scale, while this scale doesn't include a value for N-terminal acetylation, because this variant lacks the terminal positive charge (reducing polarity) it can be assumed to have increased hydrophobicity relative to FL α-syn.

Supplementary Table 2.

5’ phosphorylated primer pairs, shown in the 5’ to 3’ direction, used to mutate the FL α-syn gene.

α-Syn variant	Primer Pair	T_a / °C
5-140	Forward: aaaggactttcaaaggcc<br>Reverse: catatgtatatctccttcttaaagttaaac	60
11-140	Forward: gccaaggagggagttgtg<br>Reverse: gttctgtacaacaacctgagatcc	66
19-140	Forward: gctgagaaaaccaaacagggtgtg<br>Reverse: gttctgtacaacaacctgagatccaagc	70

a.

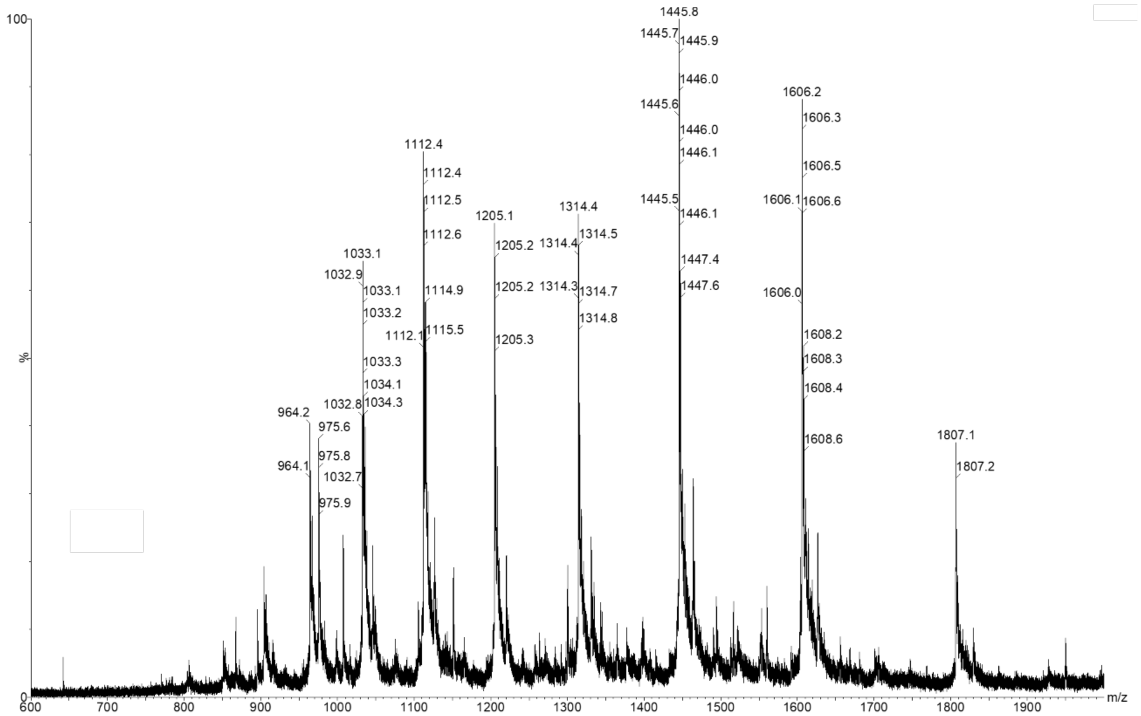

100
%
0
m/z
600
700
800
900
1000
1100
1200
1300
1400
1500
1600
1700
1800
1900
1445.8
1445.7
1445.9
1446.0
1445.6
1446.0
1446.1
1445.5
1446.1
1447.4
1447.6
1606.2
1606.3
1606.5
1606.1
1606.6
1606.0
1608.2
1608.3
1608.4
1608.6
1112.4
1112.4
1112.5
1112.6
1114.9
1112.1
1115.5
1205.1
1205.2
1205.2
1205.3
1314.4
1314.4
1314.5
1314.3
1314.7
1314.8
1033.1
1032.9
1033.1
1033.2
1033.3
1034.1
1032.8
1034.3
1032.7
964.2
964.1
975.6
975.8
975.9
1807.1
1807.2

b.

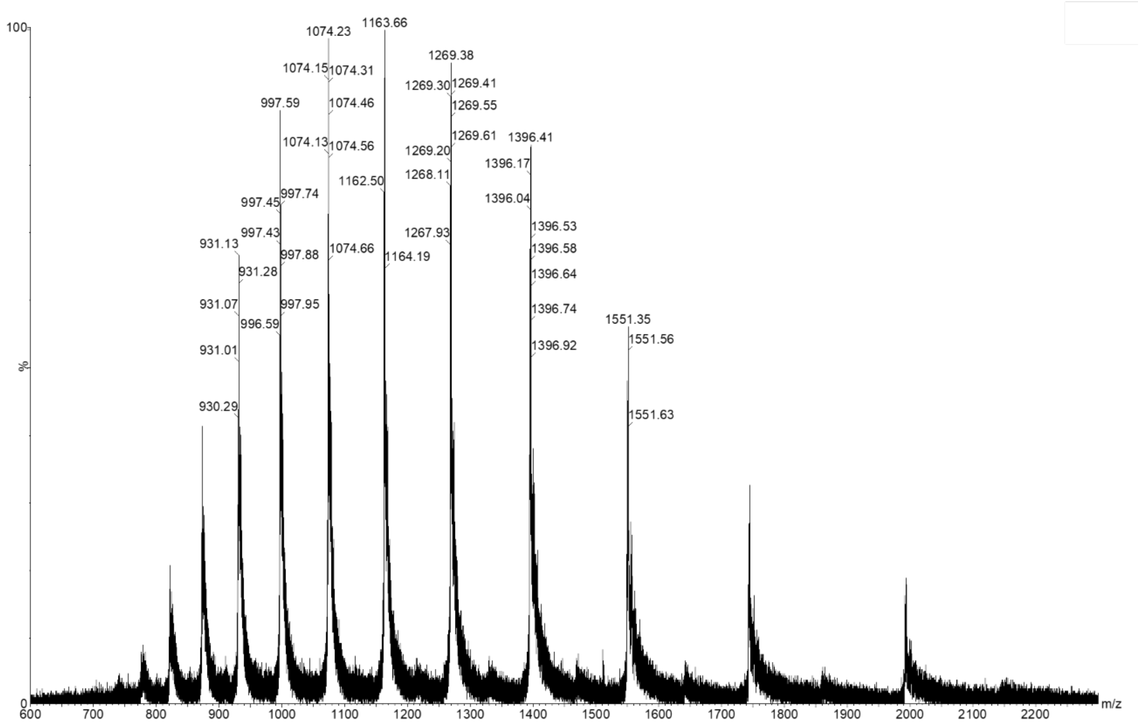

100
%
0
m/z
600
700
800
900
1000
1100
1200
1300
1400
1500
1600
1700
1800
1900
2000
2100
2200
1074.23
1163.66
1074.15
1074.31
1074.46
1074.13
1074.56
1074.66
1162.50
1164.19
997.59
997.45
997.74
997.43
997.88
997.95
996.59
931.13
931.28
931.07
931.01
930.29
1269.38
1269.30
1269.41
1269.55
1269.61
1269.20
1268.11
1267.93
1396.41
1396.17
1396.04
1396.53
1396.58
1396.64
1396.74
1396.92
1551.35
1551.56
1551.63

c.

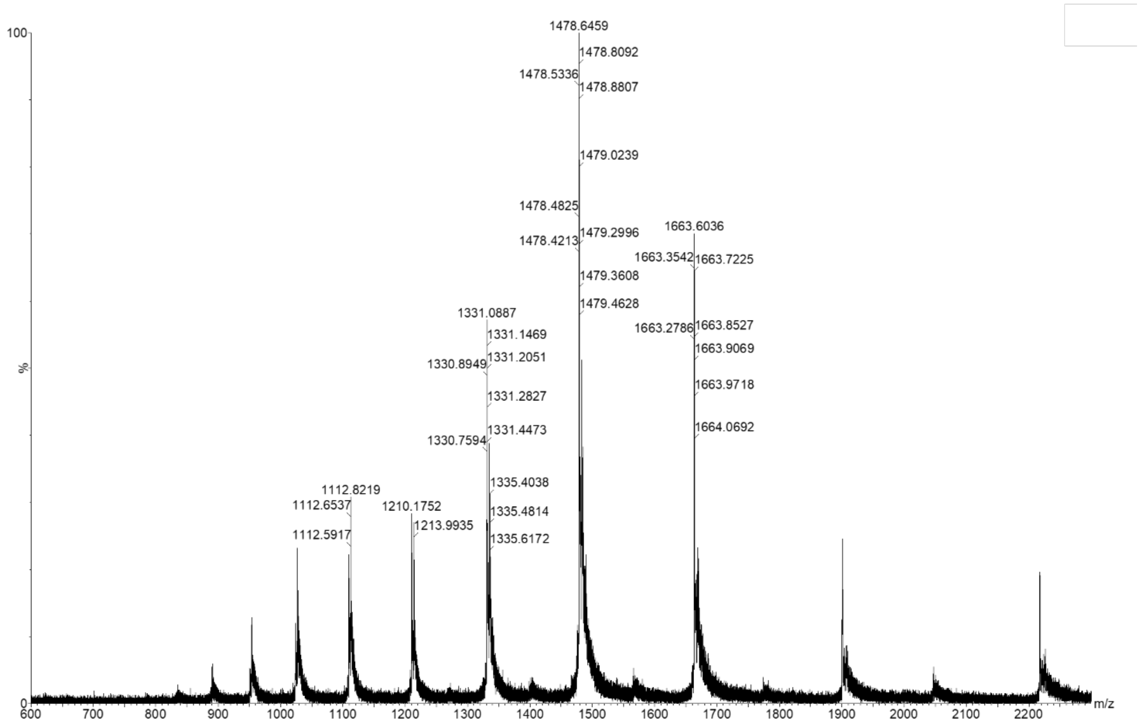
100
%
0
1478.6459
1478.8092
1478.5336
1478.8807
1479.0239
1478.4825
1478.4213
1479.2996
1479.3608
1479.4628
1663.6036
1663.3542
1663.7225
1663.2786
1663.8527
1663.9069
1663.9718
1664.0692
1331.0887
1331.1469
1330.8949
1331.2051
1331.2827
1330.7594
1331.4473
1335.4038
1335.4814
1335.6172
1112.8219
1112.6537
1112.5917
1210.1752
1213.9935
600 700 800 900 1000 1100 1200 1300 1400 1500 1600 1700 1800 1900 2000 2100 2200
m/z

d.

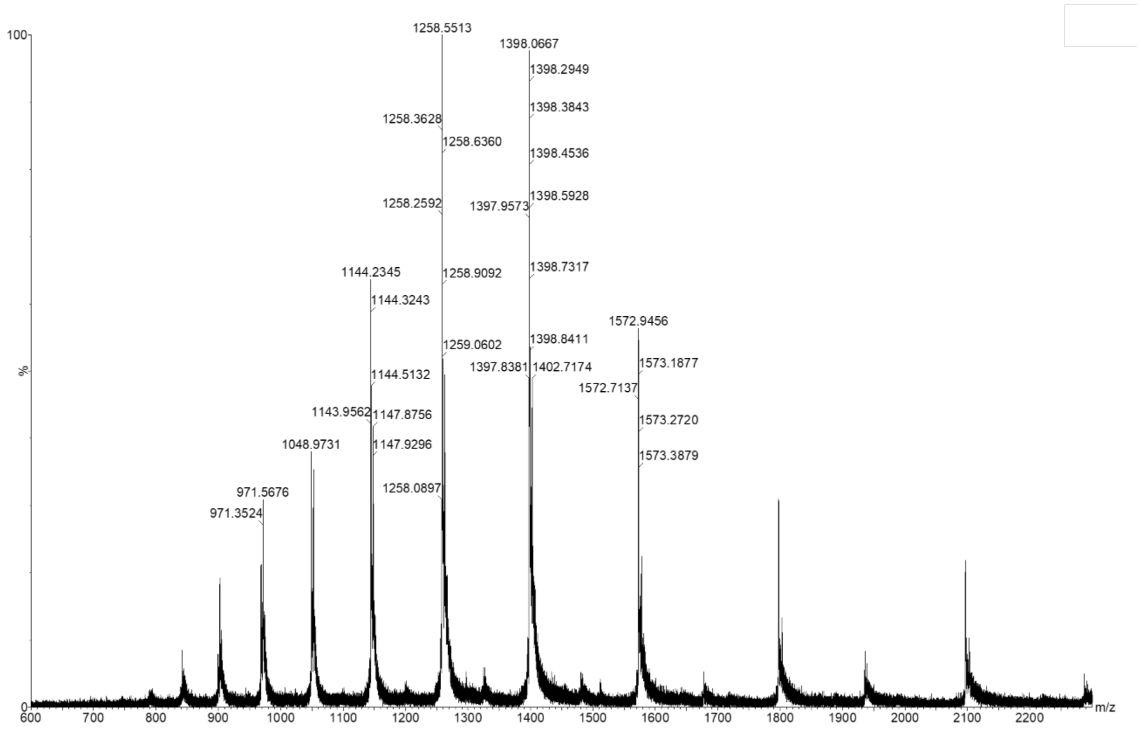
100
%
0
1258.5513
1258.3628
1258.6360
1258.2592
1258.9092
1259.0602
1258.0897
1398.0667
1398.2949
1398.3843
1398.4536
1397.9573
1398.5928
1398.7317
1398.8411
1397.8381
1402.7174
1144.2345
1144.3243
1144.5132
1143.9562
1147.8756
1147.9296
1048.9731
971.5676
971.3524
1572.9456
1573.1877
1572.7137
1573.2720
1573.3879
600 700 800 900 1000 1100 1200 1300 1400 1500 1600 1700 1800 1900 2000 2100 2200
m/z

e.

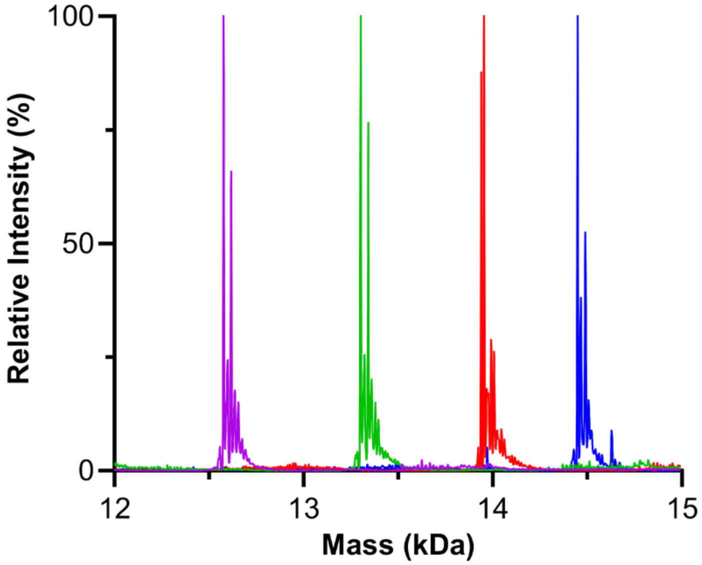

Supplementary Figure 1. N-terminally truncated α-syn purification confirmed by ESI-MS.

a-d, ESI-MS spectra of FL (a), 5-140 (b), 11-140 (c) and 19-140 (d) α-syn prior to deconvolution. e, Deconvoluted ESI-MS spectra of FL (blue), 5-140 (red), 11-140 (green) and 19-140 (purple) α-syn. Experimental and expected masses, respectively, are as follows; FL α-syn 14449.0 Da and 14451.2 Da, 5-140 α-syn 13954.0 Da and 13959.0 Da, 11-140 α-syn 13302.0 Da and 13314.6 Da, 19-140 α-syn 12577.0 Da and 12589.2 Da.

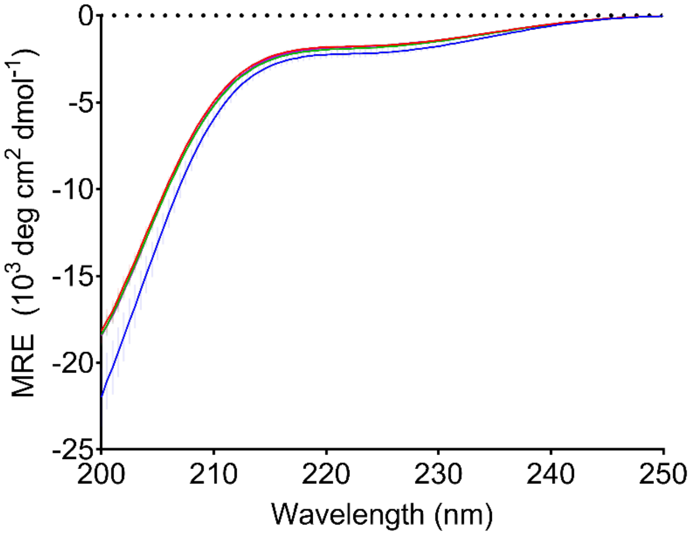

Supplementary Figure 2. N-terminally truncated α-syn retains random coil secondary structure.

Far-UV CD spectra of monomeric FL (blue), 5-140 (red), 11-140 (green) and 19-140 (purple) α-syn. The mean of three individual biological repeats is shown per sample, semi-transparent error bars represent the standard error of the mean.

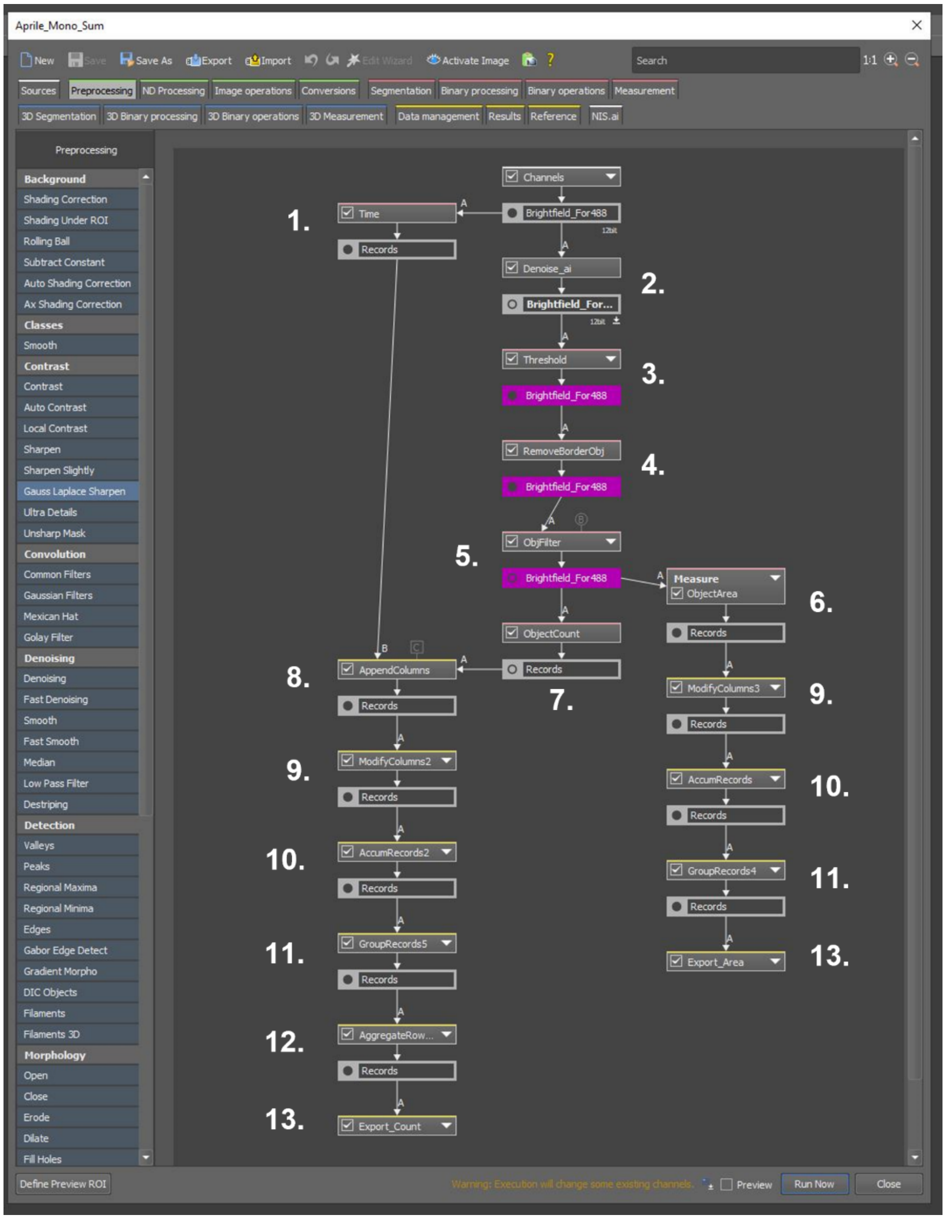

Supplementary Figure 3. GA3 recipe processes DIC images, quantifying object count and area.

Step 1 acquires the time (in s) each image was taken. Step 2 runs denoise software on the DIC images. Step 3 requires manual setting of the gain threshold range used for object identification. Step 4 reduces object misidentification by removing any objects that are touching the image boarders. Step 5 further reduces object misidentification by removing any objects that are over 50 μm^2 in area. Step 6 measures the area of every object within an image. Step 7 counts the number of objects per image. Step 8 compiles the object count and time stamp data into a single record. Step 9 removes any unwanted data from records. Step 10 repeats the object number count and object area measurements in every image taken (over every well position, z position and timepoint) and compiles them into the records. Step 11 orders and groups the records based on well position and then timepoint. Step 12 sums the total number of objects over the three z-stack images taken per well position and time point. Step 13 exports the record as a CSV file. It must be noted that this analysis was aided by visual inspection of the DIC images as this recipe cannot distinguish between condensates and other objects, e.g., dust particles. However, given the large number of condensates relative to aggregates/impurities, our quantitative analysis is robust.

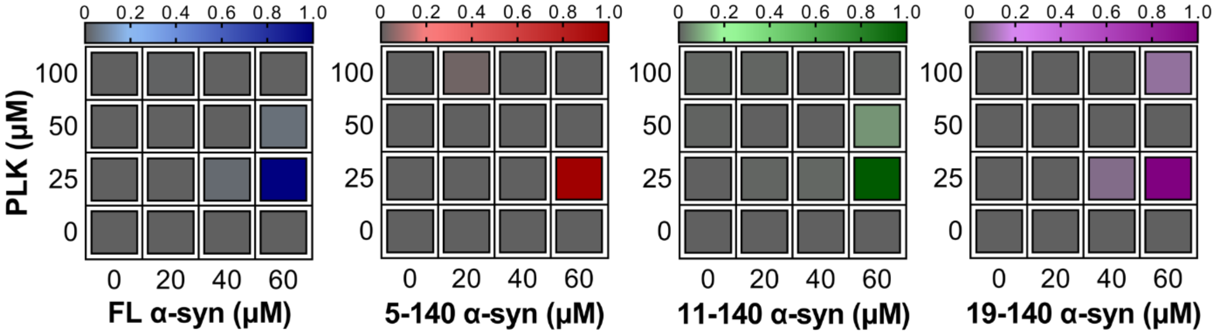

Supplementary Figure 4. N-terminal truncation does not affect α-syn condensate formation.

Representative phase diagrams showing the normalized total object count at different protein and PLK concentrations, as determined by DIC images taken at 37 °C, 15 mins after PLK addition to FL (blue), 5-140 (red), 11-140 (green), and 19-140 (purple) α-syn in phase separation buffer. Object count values for each time point are the normalized sum of the individual object counts of the three z-stack images acquired.

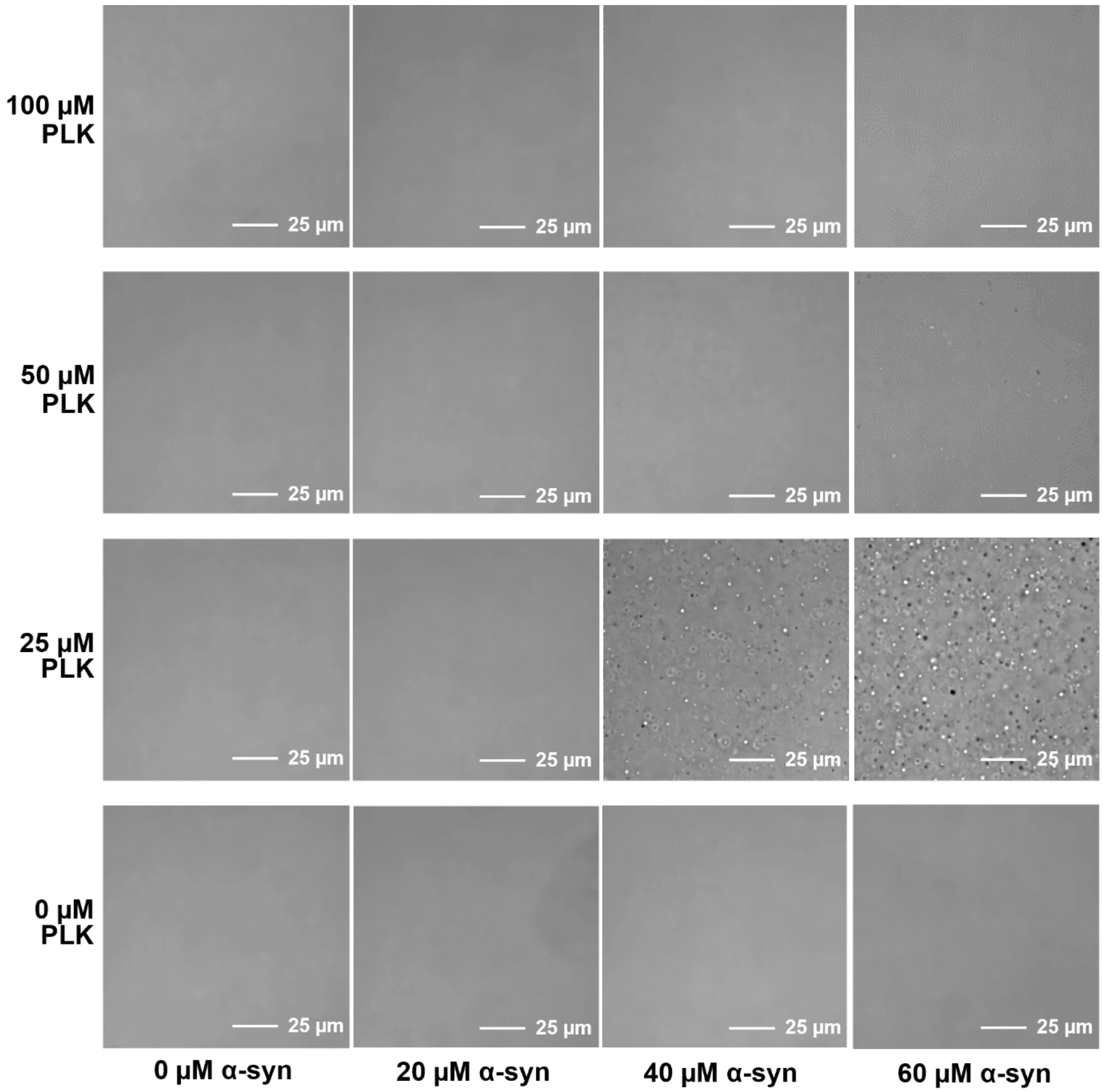

Supplementary Figure 5. Condensate formation depends on the ratio of FL α-syn to PLK.

Representative DIC images of varying concentrations of FL α-syn and PLK in phase separation buffer. Images were taken 15 mins from PLK addition at 37 °C and used to generate a phase diagram by estimating the number of condensates per image. Scale bars represent 25 µm.

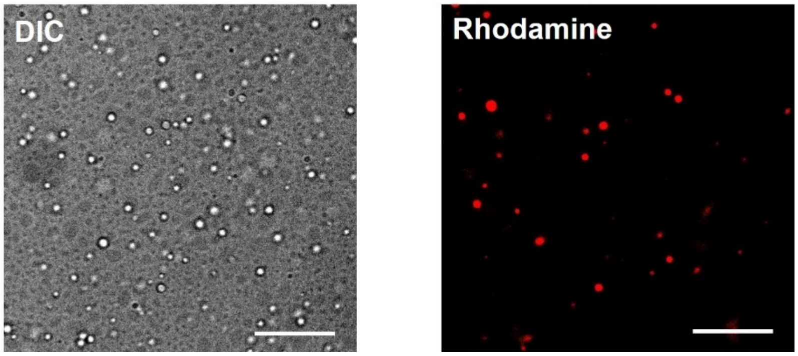

Supplementary Figure 6. α-Syn localizes within condensates.

DIC (left) and Rhodamine fluorescence (right) images of 60 μM FL α-syn incubated at 37 °C with 25 μM PLK and 1 % rhodamine labelled FL α-syn in phase separation buffer. Images were taken 30 mins after PLK addition. Scale bars represent 20 μm.

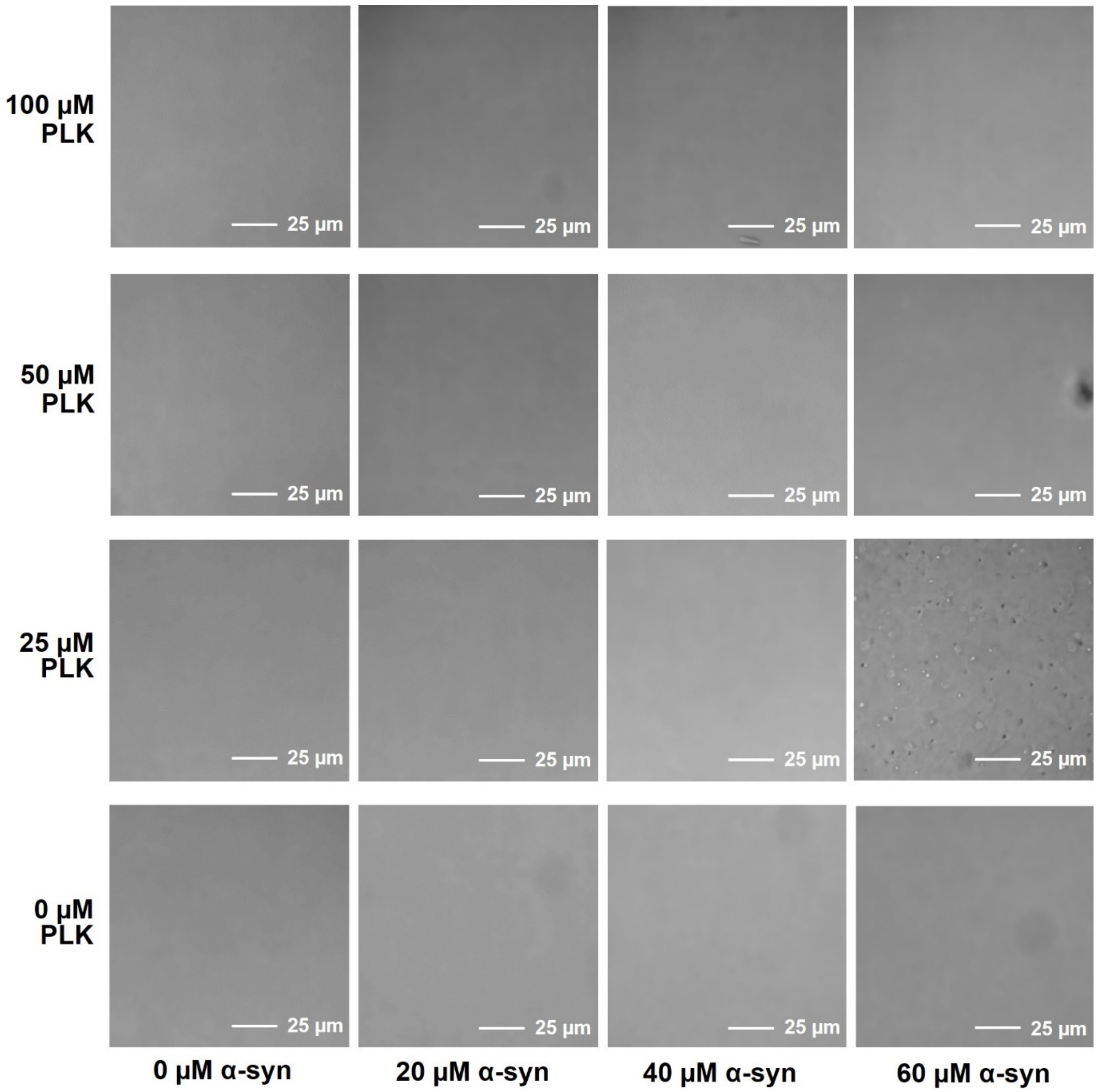

Supplementary Figure 7. 5-140 α-syn condensate formation depends on the ratio of protein to PLK.

Representative DIC images of varying concentrations of 5-140 α-syn and PLK in phase separation buffer. Images were taken 15 mins from PLK addition at 37 °C and used to generate a phase diagram by estimating the number of condensates per image. Scale bars represent 25 µm.

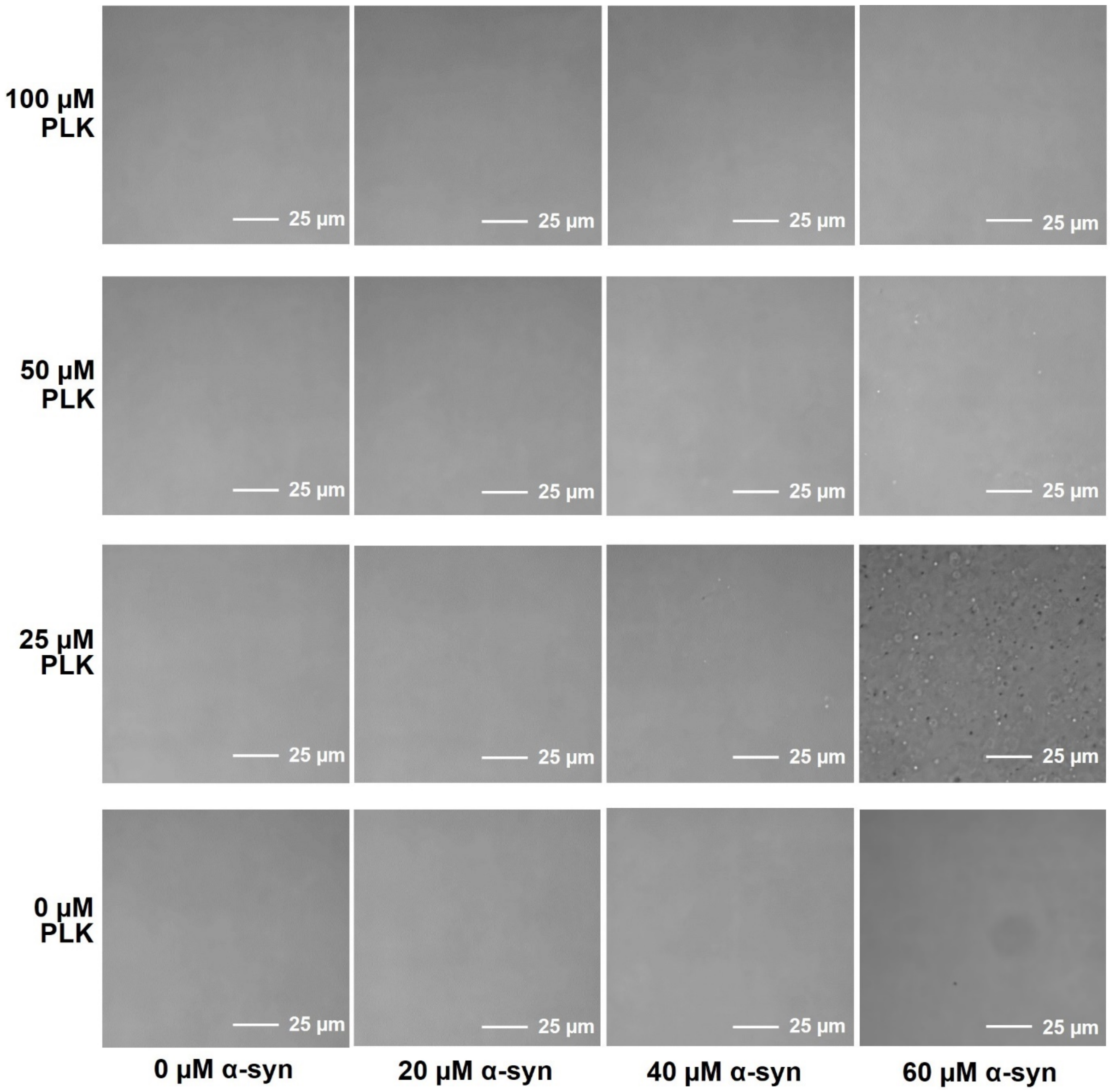

Supplementary Figure 8. 11-140 α-syn condensate formation depends on the ratio of protein to PLK.

Representative DIC images of varying concentrations of 11-140 α-syn and PLK in phase separation buffer. Images were taken 15 mins from PLK addition at 37 °C and used to generate a phase diagram by estimating the number of condensates per image. Scale bars represent 25 µm.

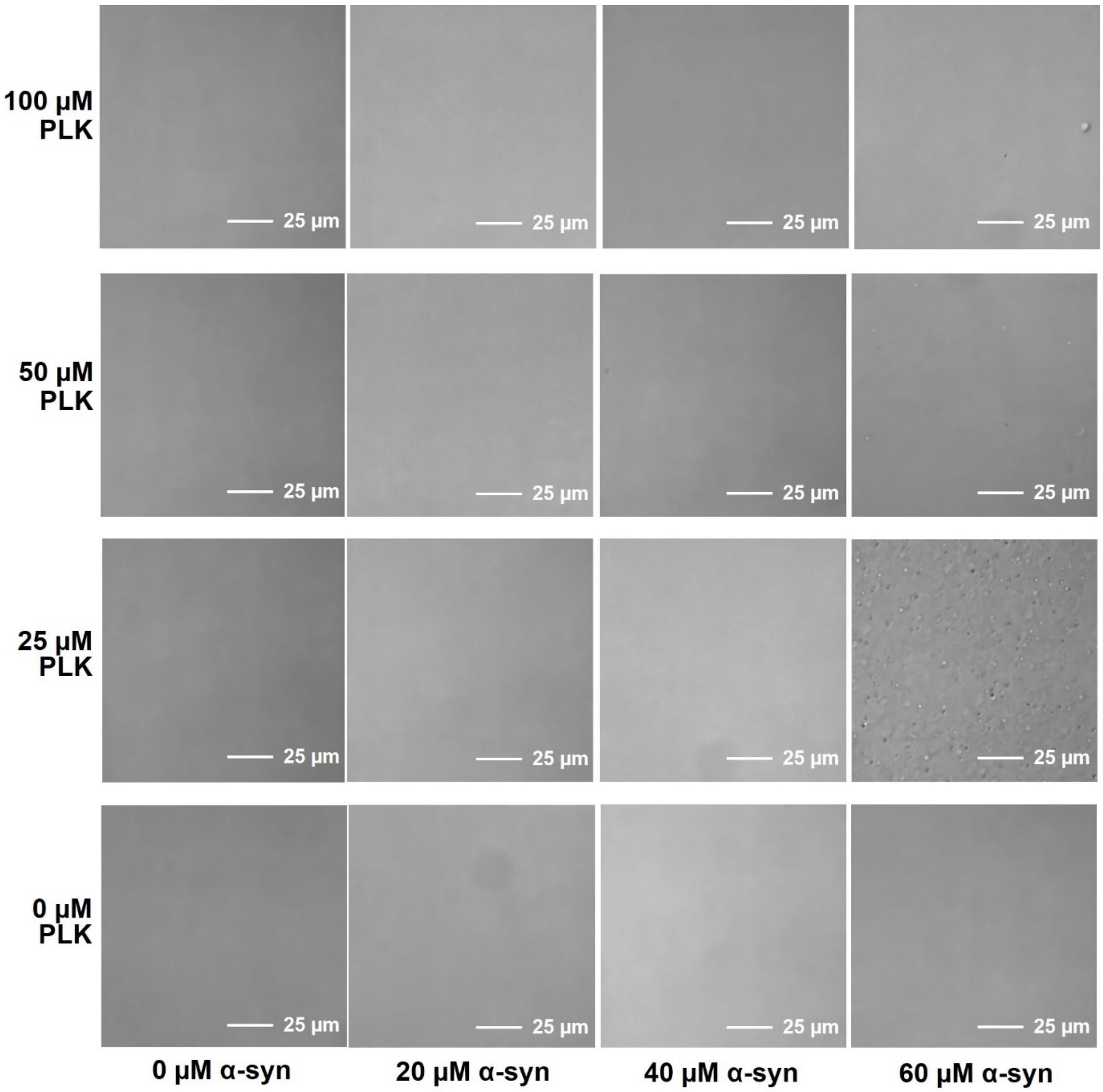

Supplementary Figure 9. 19-140 α-syn condensate formation depends on the ratio of protein to PLK.

Representative DIC images of varying concentrations of 19-140 α-syn and PLK in phase separation buffer. Images were taken 15 mins from PLK addition at 37 °C and used to generate a phase diagram by estimating the number of condensates per image. Scale bars represent 25 µm.

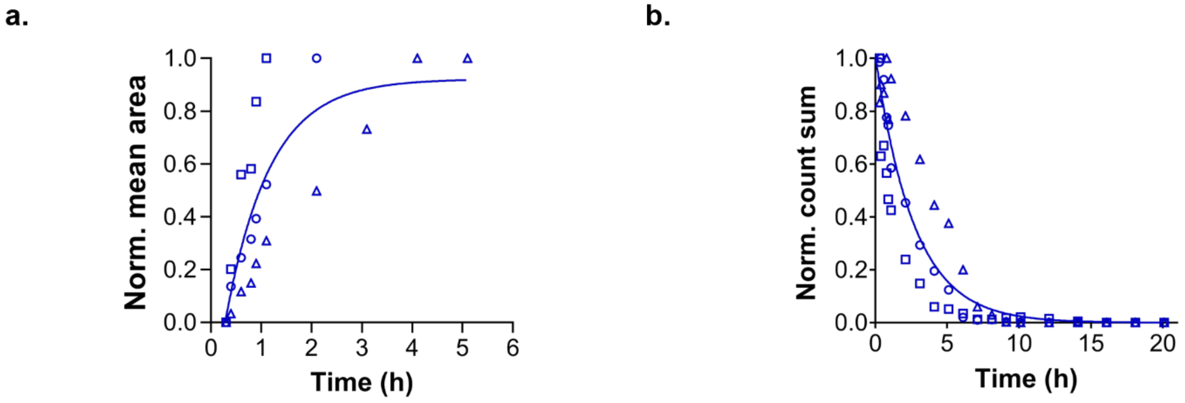

Supplementary Figure 10. FL α-syn condensates grow and reduce in number with time.

a/b, Normalized (a) mean object area and (b) total object count against time for 60 μM FL α-syn incubated at 37 °C with 25 μM PLK in phase separation buffer. Three individual biological repeats are shown, represented by circular, square or triangular symbols. Data are globally fitted with (a) a one-phase association curve or (b) a one-phase decay curve (solid lines). Mean object area values for each time point are the normalized mean of all individual object area values compiled over the three z-stack images acquired. Total object count values for each time point are the normalized sum of the individual object counts measured in each of the three z-stack images acquired.

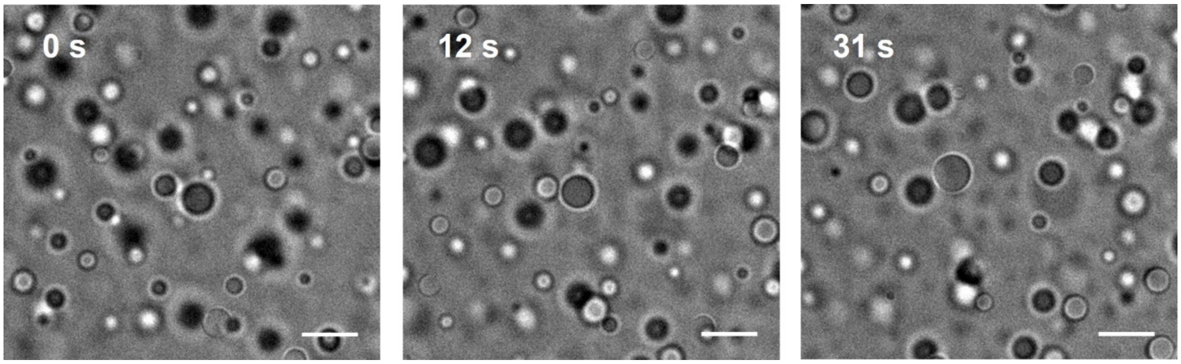

Supplementary Figure 11. Condensates can grow via coalescence.

DIC images depicting a coalescence event of two condensates in solution for 60 μM FL α-syn with 25 μM PLK. Images were taken ~ 20 mins after PLK addition using a 60x oil objective. Scale bars represent 10 μm.

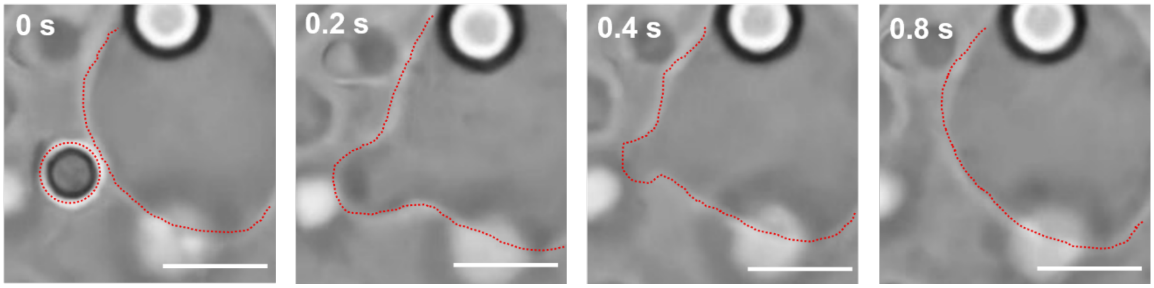

Supplementary Figure 12. Surface-wetted condensates can grow via coalescence.

DIC images manually acquired at the bottom of the well after ~ 1.5 h incubation of 60 μM FL α-syn with 25 μM PLK. Images depict a coalescence event (scale bars represent 10 μm).

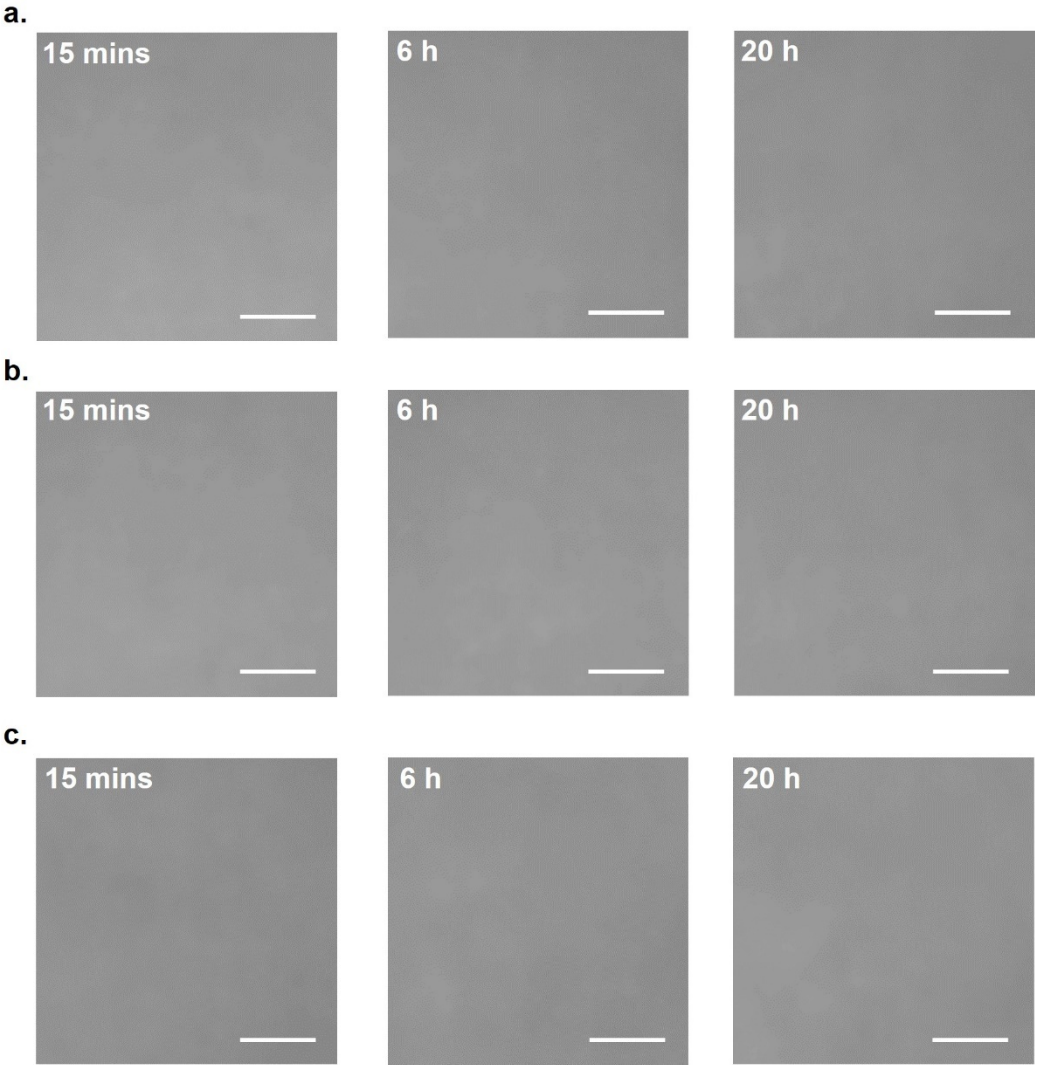

Supplementary Figure 13. Control samples do not form soluble condensates or aggregates.

Representative DIC images of (a) phase separation buffer, (b) 25 μM PLK and (c) 60 μM FL α-syn at selected time points. Scale bars represent 25 μm.

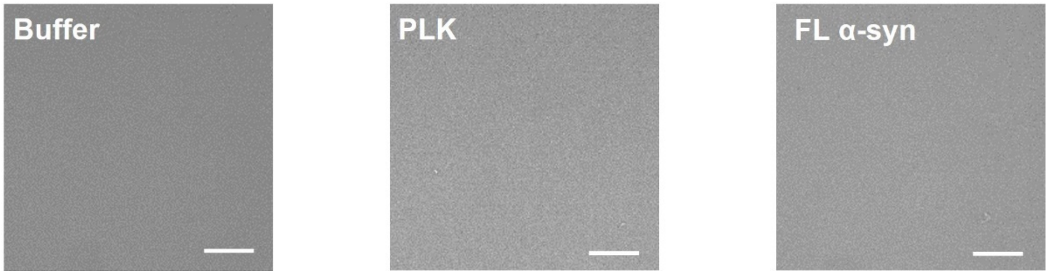

Supplementary Figure 14. Control samples do not form sedimented condensates or aggregates.

Representative DIC images taken at the bottom surface of the well after 20 h incubation at 37 °C in phase separation buffer. Control samples of buffer, 25 μM PLK or 60 μM FL α-syn are shown. Scale bars represent 25 μm.

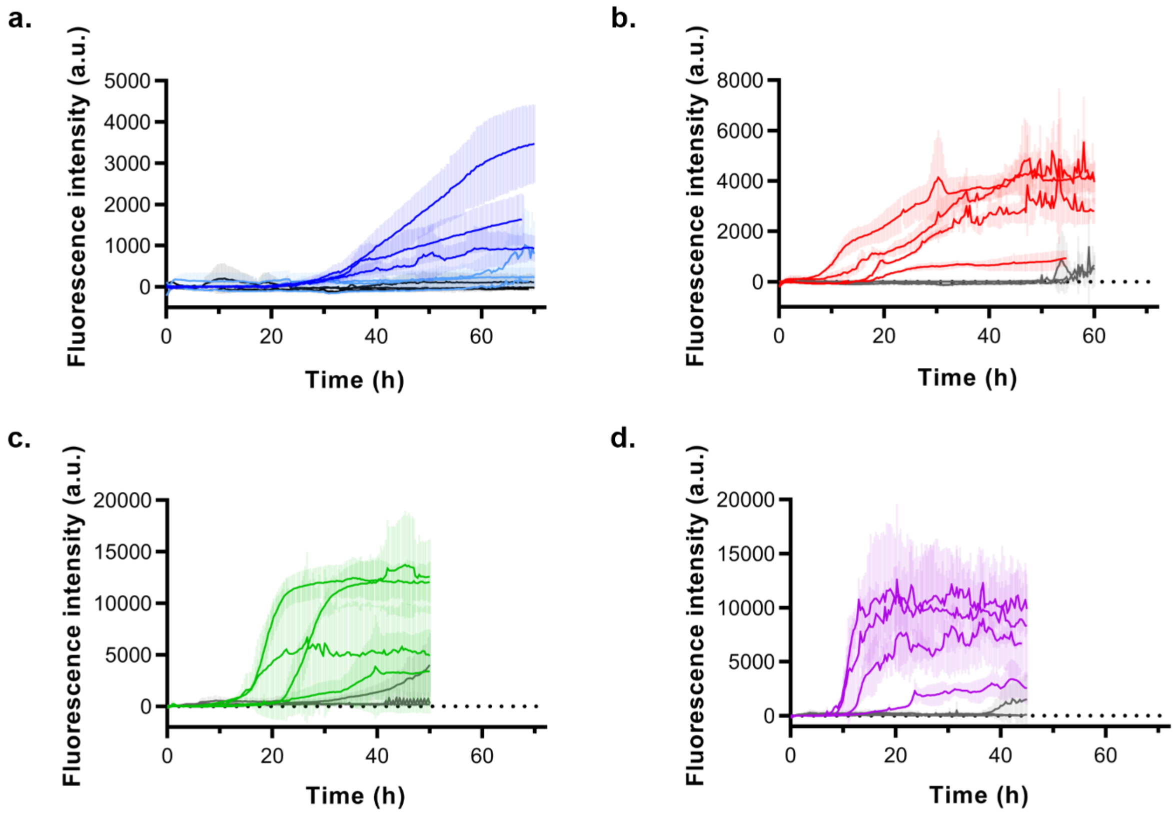

Supplementary Figure 15. N-terminal truncation accelerates aggregation following α-syn phase separation.

a, Raw ThT fluorescence intensity data when 60 μM FL α-syn with 25 μM PLK (dark blue), 60 μM FL α-syn (light blue) and 25 μM PLK (grey), are incubated at 37 °C in phase separation buffer. b-d, Raw ThT fluorescence intensity data when 5-140 (red), 11-140 (green) or 19-140 (purple) α-syn are incubated in phase separation buffer at 37 °C. 60 μM α-syn alone (grey) is also shown for each variant. Each repeat is the mean of three technical replicates and semi-transparent error bars represent the standard deviation of the mean.

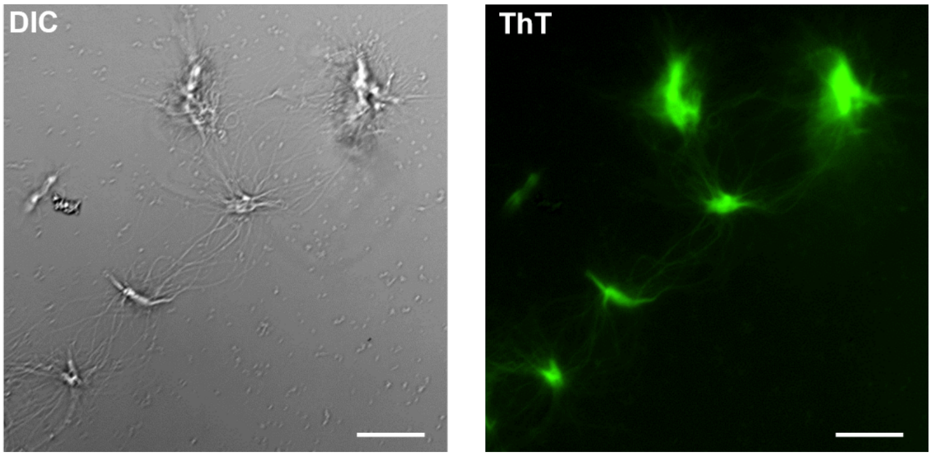

Supplementary Figure 16. N-terminal truncation accelerates aggregation following α-syn phase separation.

Representative DIC (left) and fluorescent (right) images of 60 μM FL α-syn with 25 μM PLK at the endpoint of a phase separation ThT aggregation assay. Images are of the bottom of the well, scale bars represent 25 μm.

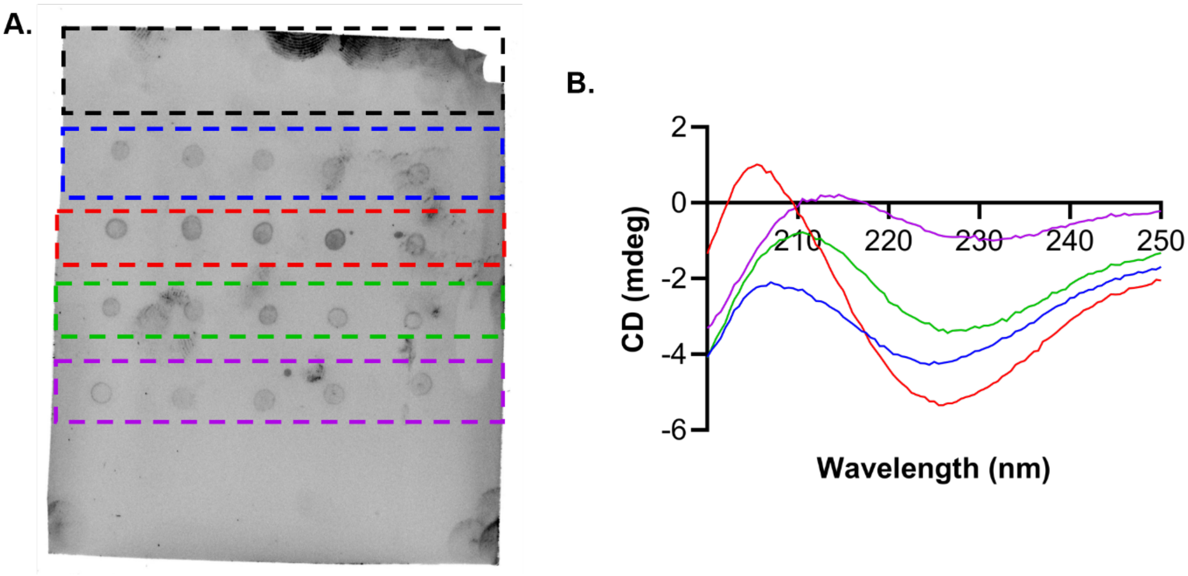

Supplementary Figure 17. Aggregates formed under phase separation conditions contain α-syn and β-sheet secondary structure.

a, Representative dot-blot analysis of the insoluble aggregate pellet formed when FL (blue), 5-140 (red), 11-140 (green) and 19-140 (purple) α-syn were aggregated under phase separation conditions. As a control, analysis was also performed on a sample of 25 μM PLK in PBS (black), no fluorescence signal was observed for this sample confirming the specificity of the anti-α-syn primary antibody to α-syn. 5 replicates are shown per sample. b, Far-UV CD spectra of the insoluble aggregate pellet formed when FL (blue), 5-140 (red), 11-140 (green) and 19-140 (purple) α-syn were aggregated under phase separation conditions. The mean of five accumulations is shown per sample. As the aggregates formed under phase separation conditions may contain unknown concentrations of PLK, alongside α-syn, it was not possible to convert to MRE and so the raw data are shown here.

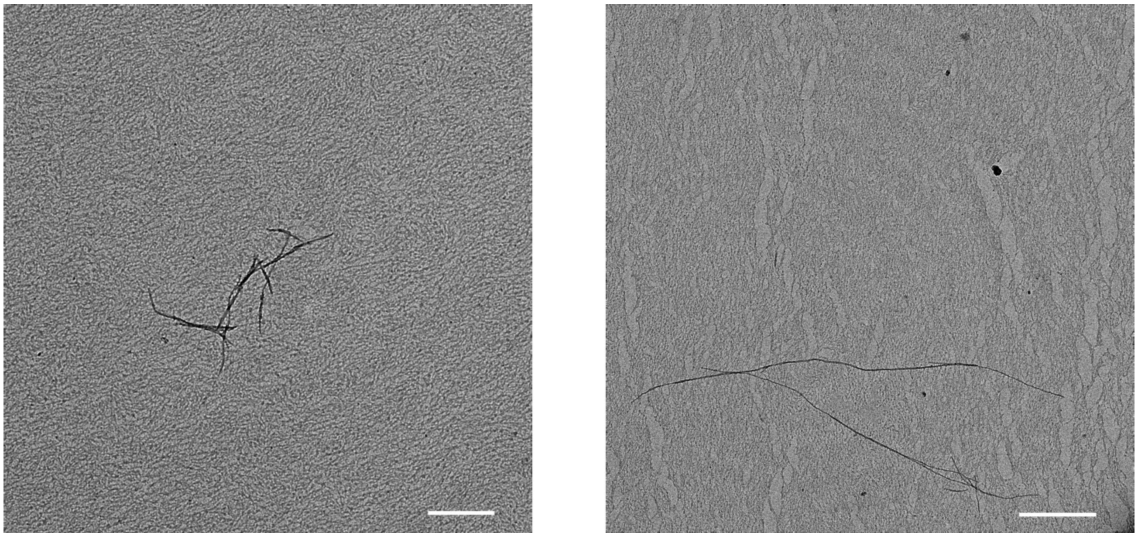

Supplementary Figure 18. α-syn aggregation is delayed in the absence of phase separation.

Representative TEM images of 60 μM FL α-syn aggregated alone (i.e., in the absence of PLK) in phase separation buffer supplemented with 20 μM ThT. An aliquot of the whole sample was taken at the endpoint of a phase separation ThT aggregation assay and applied to a TEM grid. Scale bars represent 500 nm (left) and 1 μm (right).

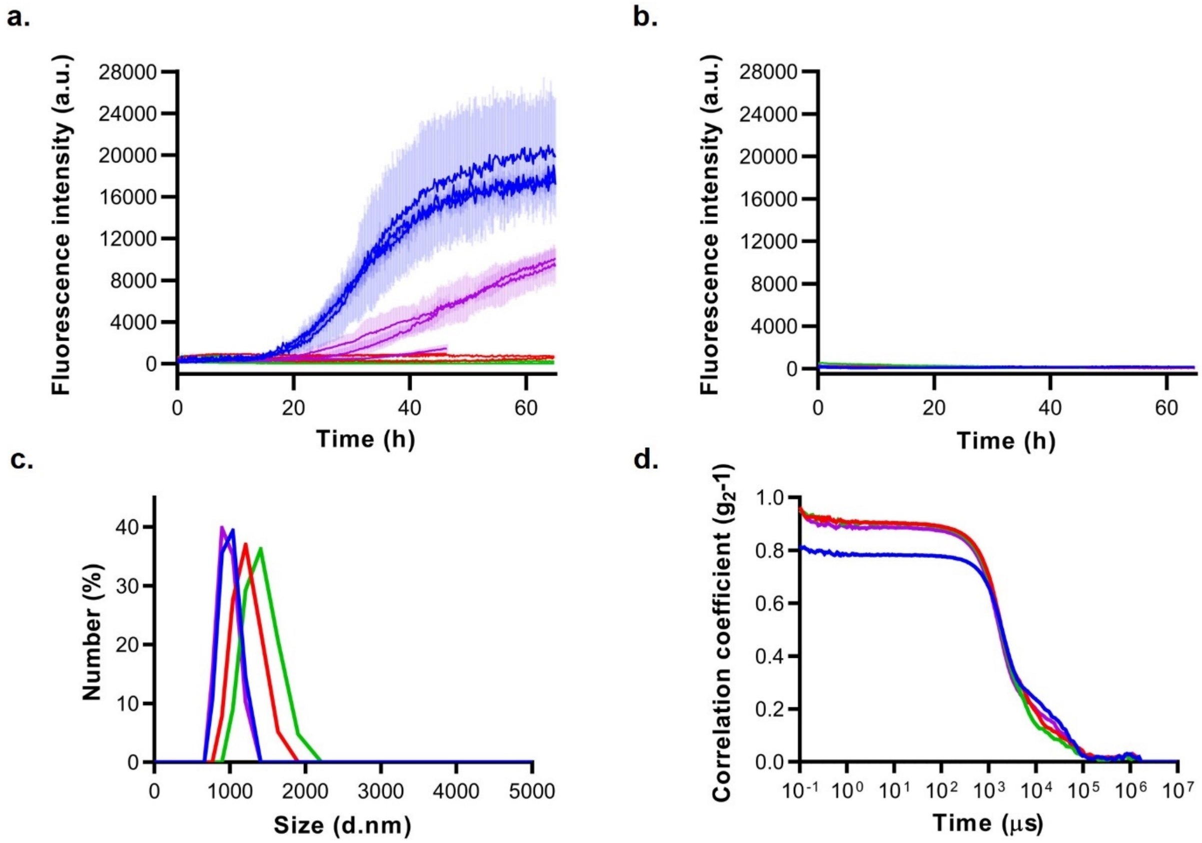

Supplementary Figure 19. N-terminal truncation delays elongation of α-syn fibrils formed via phase separation.

a/b, Elongation kinetics of FL (blue), 5-140 (red), 11-140 (green) and 19-140 (purple) α-syn fibrils formed under via separation, either in the presence (a) or absence (b) of the corresponding monomeric protein. Three biological repeats are shown. Each repeat is the mean of three technical replicates, semi-transparent error bars represent the standard deviation. c/d, Representative number distribution (c) and the corresponding correlogram (d) of FL (blue), 5-140 (red), 11-140 (green) and 19-140 (purple) α-syn fibrils formed via phase separation and used to induce fibril elongation.

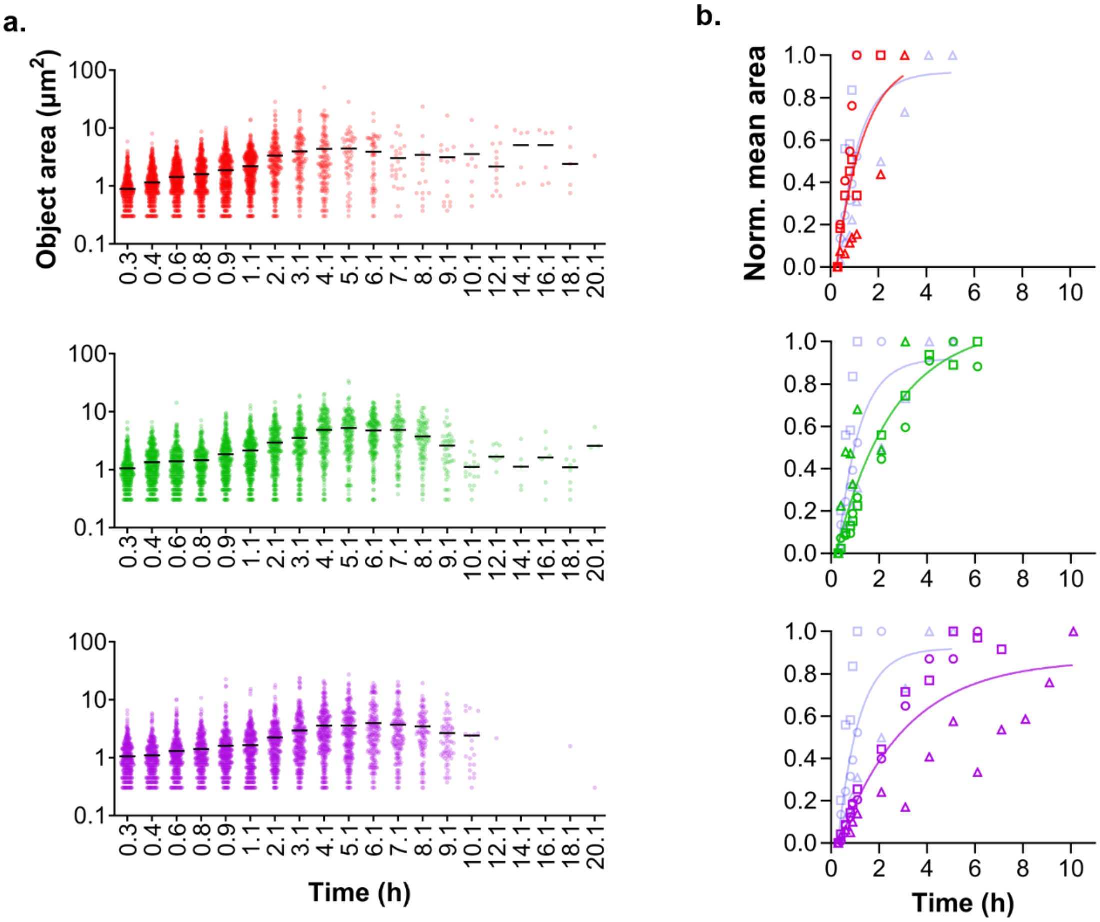

Supplementary Figure 20. N-terminal truncation increases α-syn condensate lifetime in solution.

a, Object area distribution over time for 5-140 (red), 11-140 (green) and 19-140 (purple) α-syn. Each timepoint was compiled over the three z-stack images. Mean droplet areas at each time point are indicated by a solid black line. b, Normalized mean object area against time for 60 µM 5-140 (red), 11-140 (green) and 19-140 (purple) α-syn incubated with 25 µM PLK in phase separation buffer. Three individual biological repeats are shown, represented by circular, square or triangular symbols. The data is globally fit with a one-phase association curve (solid line). Mean object area values for each time point are the normalized mean of all individual object area values compiled over the three z-stack images acquired. Each plot is overlayed with the corresponding FL α-syn data, shown in semi-transparent blue, for comparison.

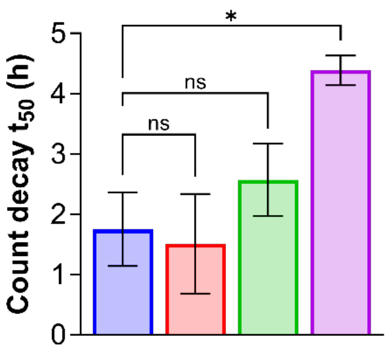

Supplementary Figure 21. Increasing N-terminal truncation increases α-syn condensate lifetime in solution.

Half-life (t50) of the decay in object count for 60 μM FL (blue), 5-140 (red), 11-140 (green) and 19-140 (purple) α-syn with 25 μM PLK in phase separation buffer. Error bars represent the standard error of the mean for three biological repeats.

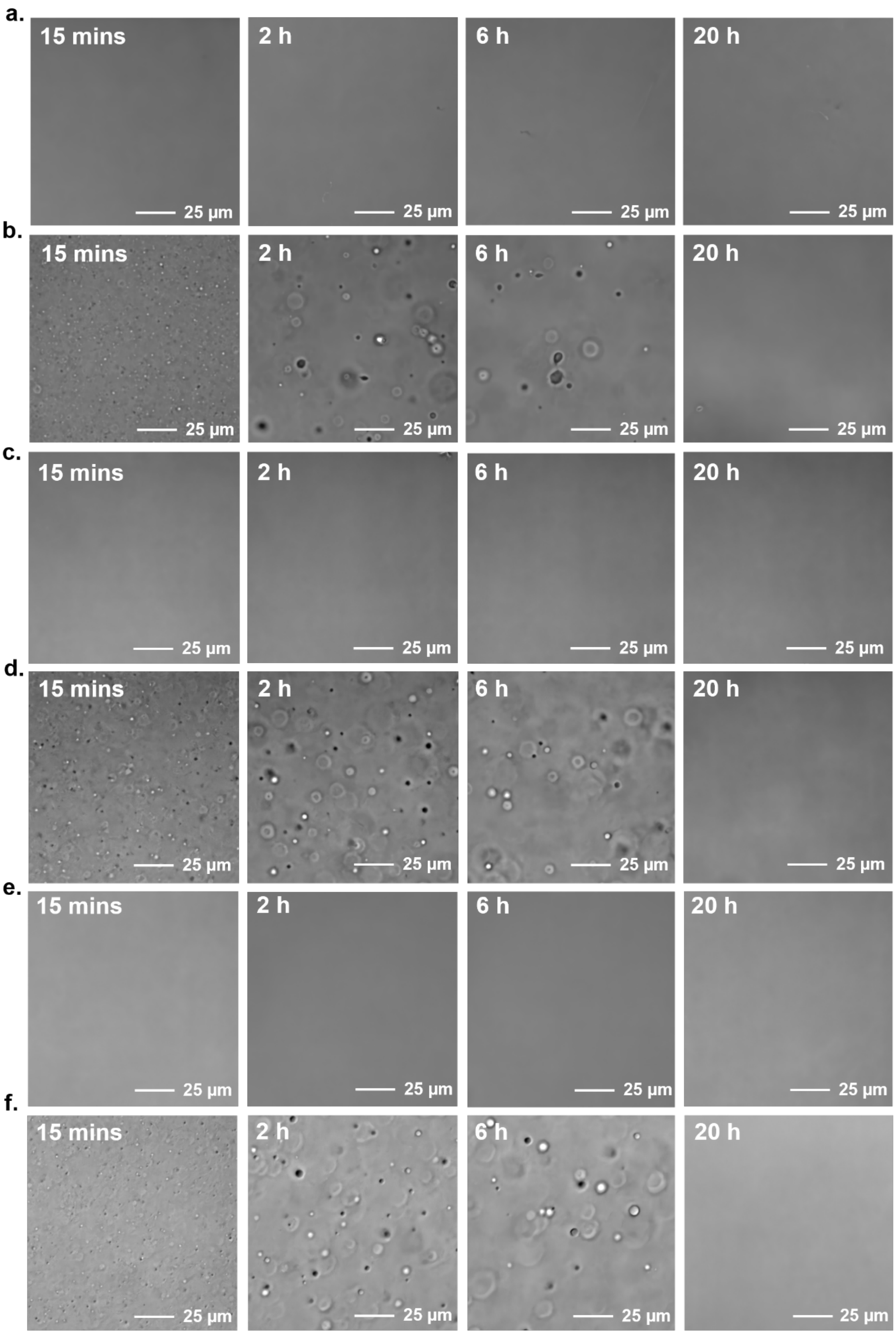
a.
15 mins
2 h
6 h
20 h
25 µm
b.
c.
d.
e.
f.

Supplementary Figure 22. N-terminally truncated α-syn condensates grow and decrease in number over time.

Representative DIC images of (a) 60 μM 5-140 α-syn, (b) 60 μM 5-140 α-syn with 25 μM PLK, (c) 60 μM 11-140 α-syn, (d) 60 μM 11-140 α-syn with 25 μM PLK, (e) 60 μM 19-140 α-syn, and (f) 60 μM 19-140 α-syn with 25 μM PLK. Selected time points are shown. Scale bars represent 25 μm.

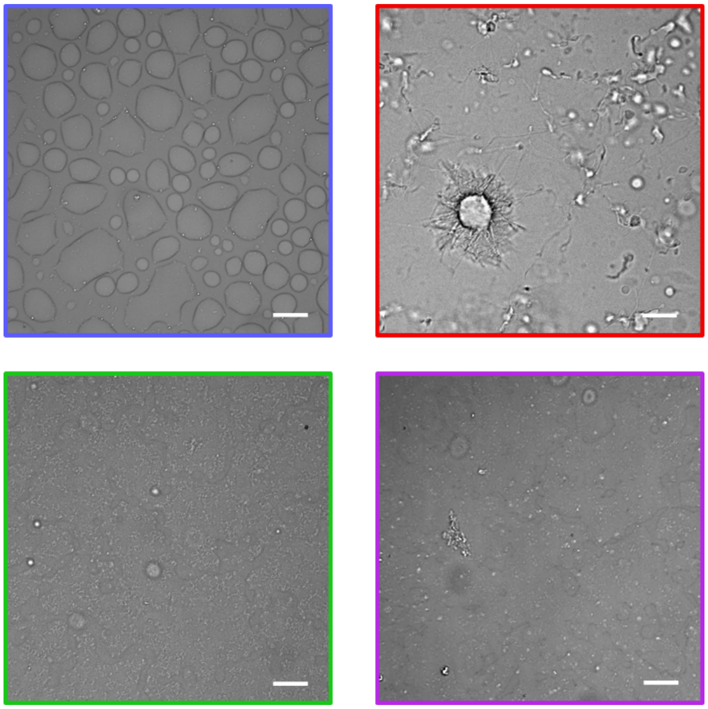

Supplementary Figure 23. N-terminally truncated α-syn condensates grow and decrease in number over time.

Representative DIC images acquired manually at the bottom of the well after 20 h incubation of FL (blue), 5-140 (red), 11-140 (green) and 19-140 (purple) α-syn under phase separation conditions (scale bars represent 25 μm).

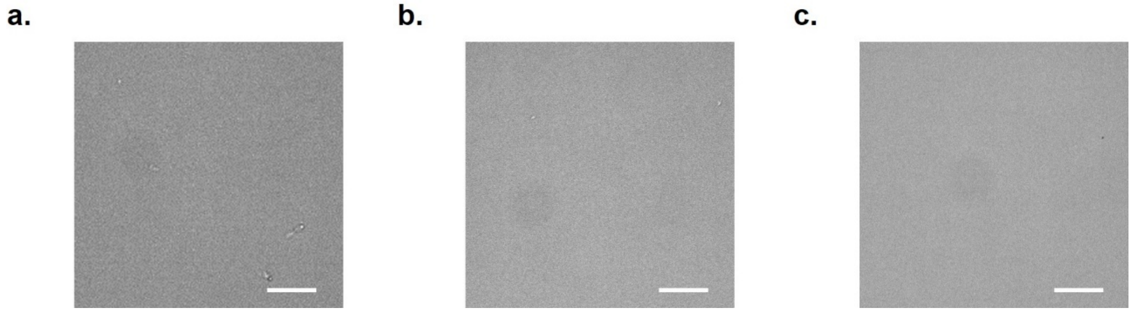

Supplementary Figure 24. N-terminally truncated α-syn alone does not form sedimented condensates or aggregates.

Representative DIC images taken at the bottom of the well after 20 h incubation of 60 μM (a) 5-140, (b) 11-140, and (c) 19-140 α-syn at 37 °C in phase separation buffer. Scale bars represent 25 μm.

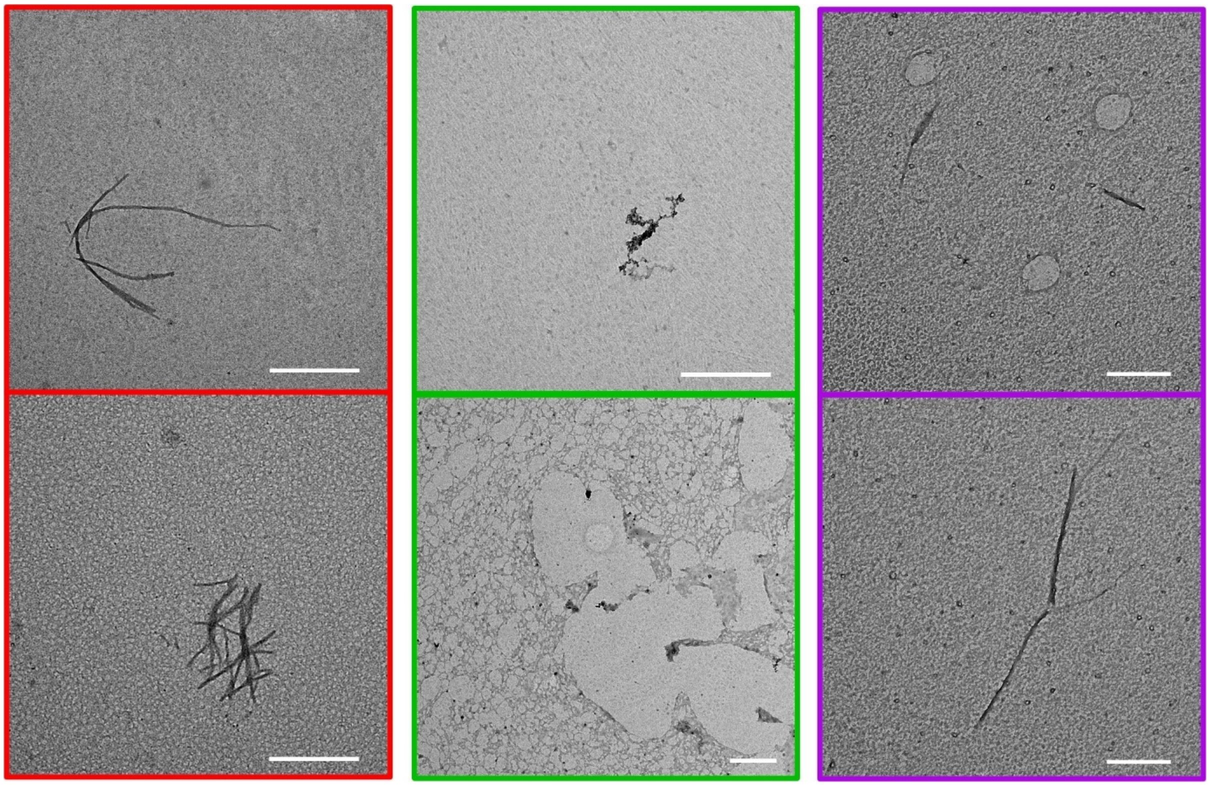

Supplementary Figure 25. N-terminally truncated α-syn aggregation is delayed in the absence of phase separation.

Representative TEM images of 60 μM 5-140 (red), 11-140 (green) and 19-140 (purple) α-syn aggregated alone (i.e., in the absence of PLK) in phase separation buffer with 20 μM ThT. An aliquot of the whole sample was taken at the endpoint of a phase separation ThT aggregation assay and applied to the TEM grid. Scale bars represent 500 nm.

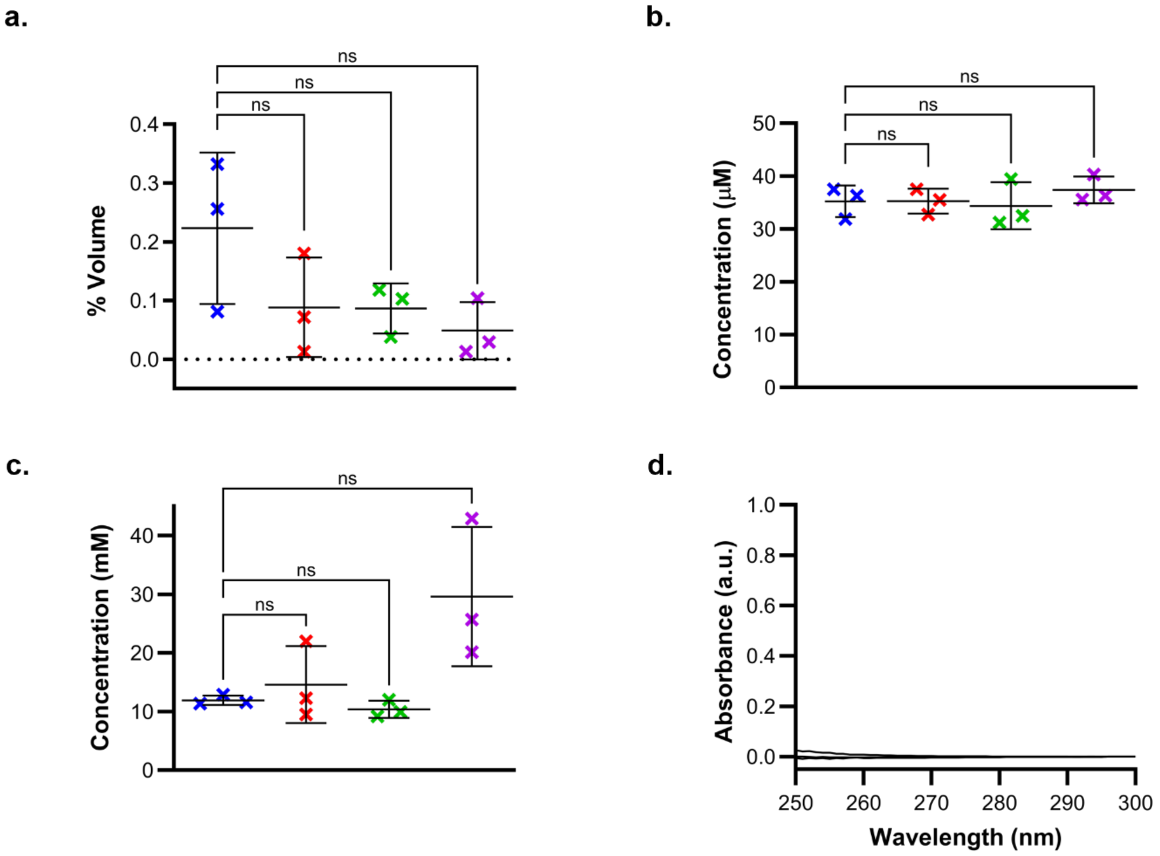

Supplementary Figure 26. N-terminal truncation does not significantly alter phase partitioning.

a, Volume fraction of FL (blue), 5-140 (red), 11-140 (green) and 19-140 (purple) α-syn condensates. b/c, Concentration of FL (blue), 5-140 (red), 11-140 (green) and 19-140 (purple) α-syn in the dilute-phase (b) and the dense-phase (c). d. Supernatant absorbance after centrifugation of 25 μM PLK in phase separation buffer. Three biological replicates are shown, the spectra have been buffer subtracted and baseline corrected at 300 nm.

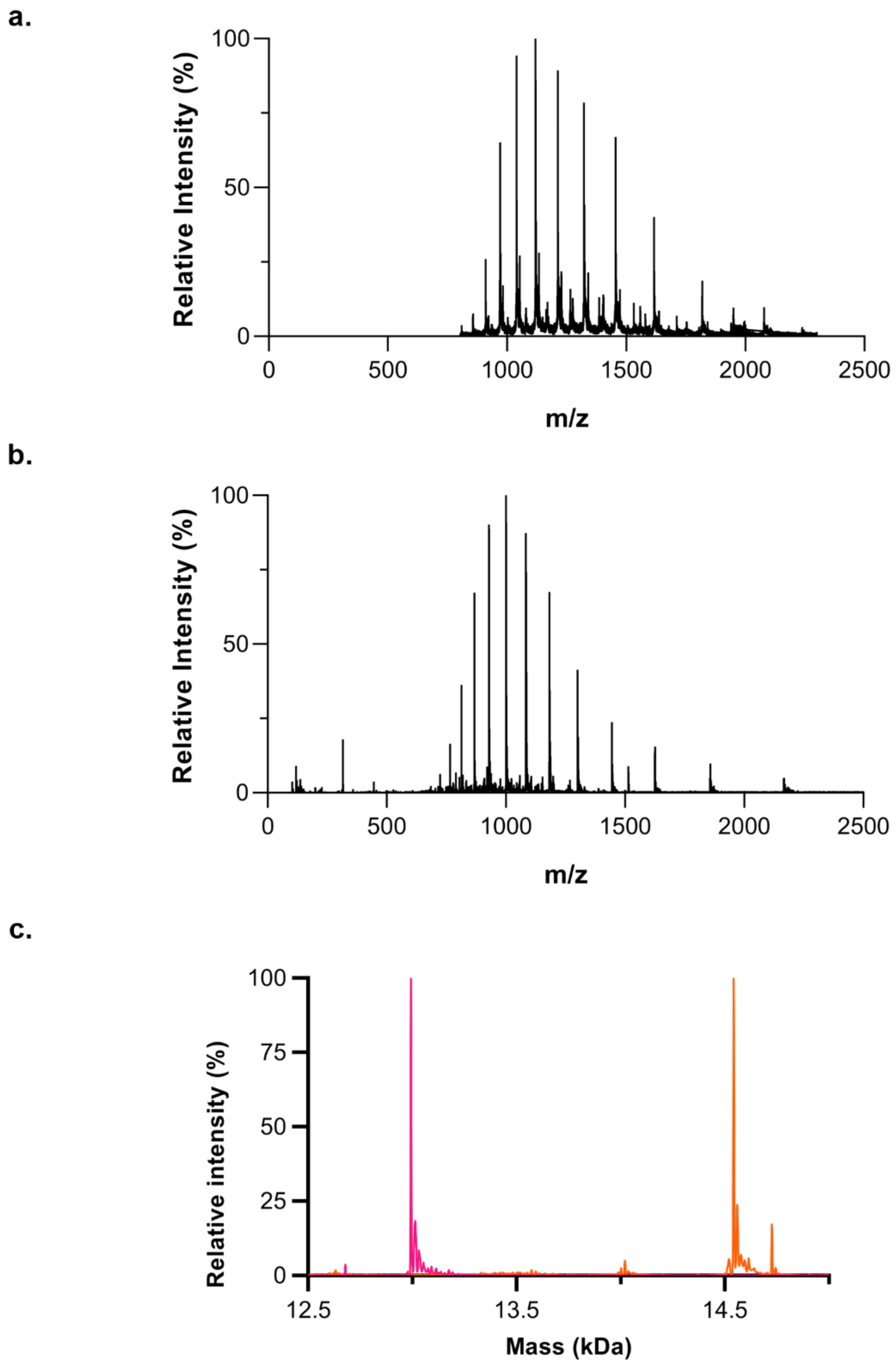

Supplementary Figure 27. N-terminally modified α-syn purification confirmed by ESI-MS.

a/b, ESI-MS spectra of AcFL (a) and 14-140 (b) α-syn prior to deconvolution. c, Deconvoluted ESI-MS spectra of AcFL (orange) and 14-140 (pink) α-syn. Experimental and expected masses, respectively, are as follows; AcFL α-syn 14543 Da and 14531 Da, 14-140 α-syn 12995 Da and 13022 Da.

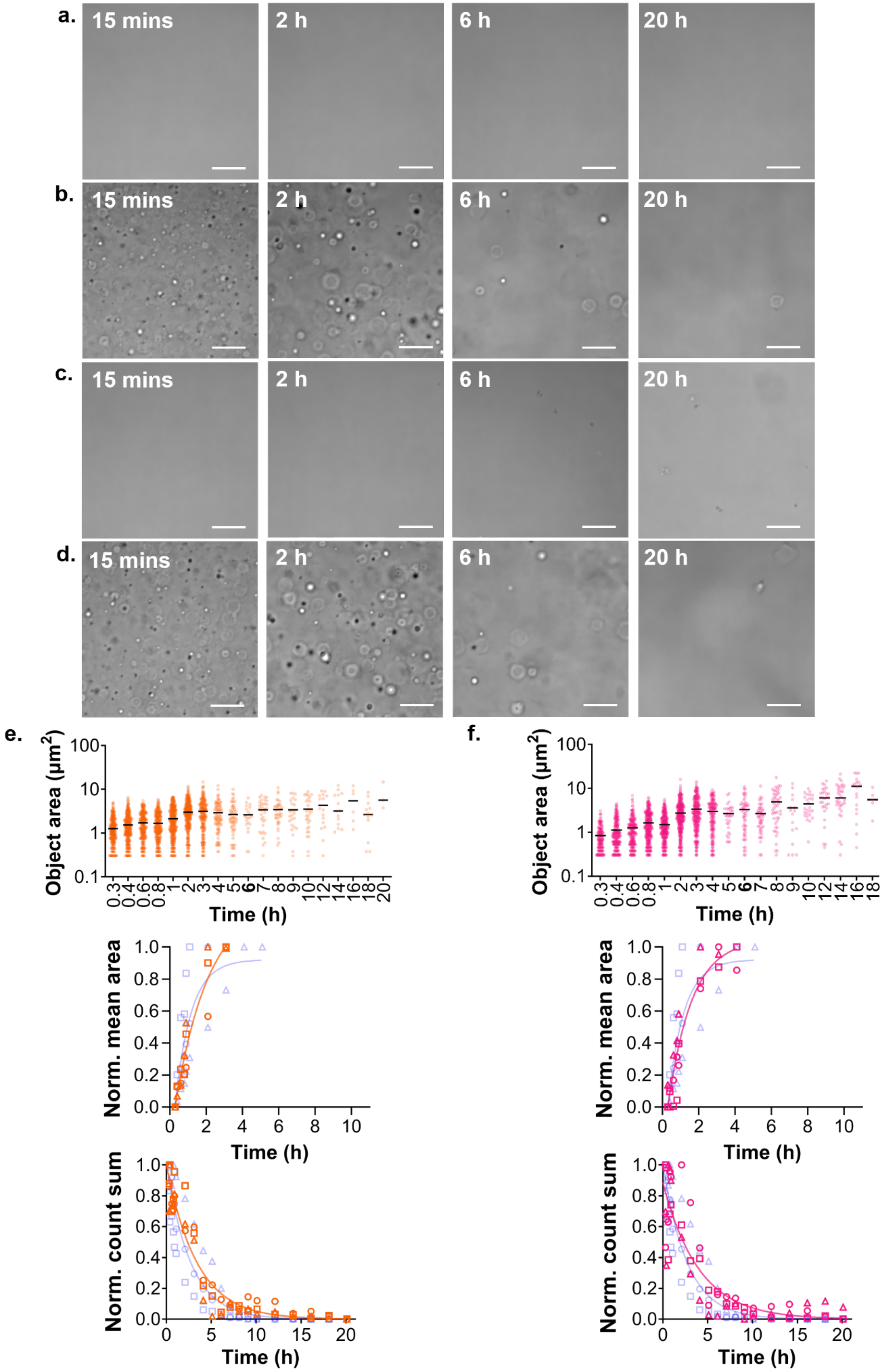
a.
15 mins
2 h
6 h
20 h
b.
15 mins
2 h
6 h
20 h
c.
15 mins
2 h
6 h
20 h
d.
15 mins
2 h
6 h
20 h
e.
Object area (μm²)
Time (h)
Norm. mean area
Time (h)
Norm. count sum
Time (h)
f.
Object area (μm²)
Time (h)
Norm. mean area
Time (h)
Norm. count sum
Time (h)

Supplementary Figure 28. N-terminally modified α-syn condensates grow and sediment over time.

a-d. Representative DIC images of (a) 60 μM AcFL α-syn, (a) 60 μM AcFL α-syn with 25 μM PLK, (c) 60 μM 14-140 α-syn, and (d) 60 μM 14-140 α-syn with 25 μM PLK. Selected time points are shown. Scale bars represent 25 μm. e/f. Condensate maturation data for 60 μM AcFL (e) or 14-140 (f) α-syn incubated at 37 °C with 25 μM PLK in phase separation buffer. Top, representative object area distribution over time. Each timepoint was compiled over the three z-stack images. Mean droplet areas at each time point are indicated by a solid black line. Normalized mean object area (middle) and total object count (bottom) against time. Three biological repeats are shown, represented by circular, square or triangular symbols. Data are globally fitted with a one-phase association curve (middle) or a one-phase decay curve (bottom). Mean object area values are the normalized mean of all object area values compiled over the three z-stack images acquired. Total object count values are the normalized sum of the individual object counts for the three z-stack images acquired. Both plots are overlayed with the corresponding FL α-syn data, shown in semi-transparent blue.

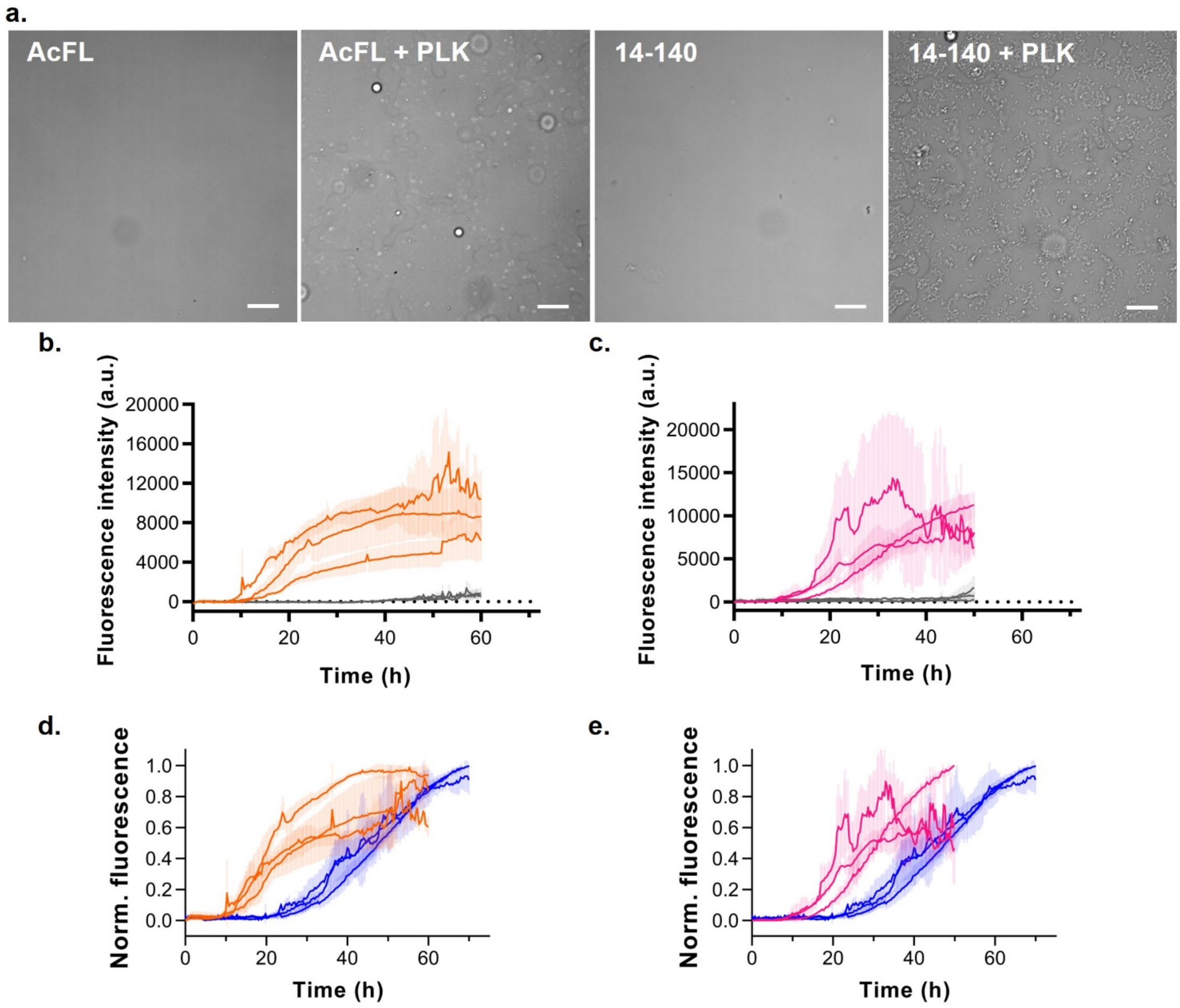

Supplementary Figure 29. N-terminally modified α-syn condensates modulate surface wetting and aggregation.

a. Representative DIC images acquired manually at the bottom of the well after 20 h incubation of AcFL and 14-140 α-syn in the presence or absence of PLK (scale bars represent 25 μm). b/c, Raw ThT fluorescence intensity data when AcFL (orange) or 14-140 (pink) α-syn are incubated with 25 μM PLK in phase separation buffer at 37 °C. 60 μM α-syn alone (grey) is also shown for each variant. Each repeat is the mean of three technical replicates and semi-transparent error bars represent the standard deviation of the mean. d/e, Corresponding normalized ThT intensity data for AcFL (orange) or 14-140 (pink) α-syn incubated with 25 μM PLK, alongside the FL α-syn data (blue) for comparison. Each repeat is the mean of three technical replicates, error bars represent the standard deviation of the mean.

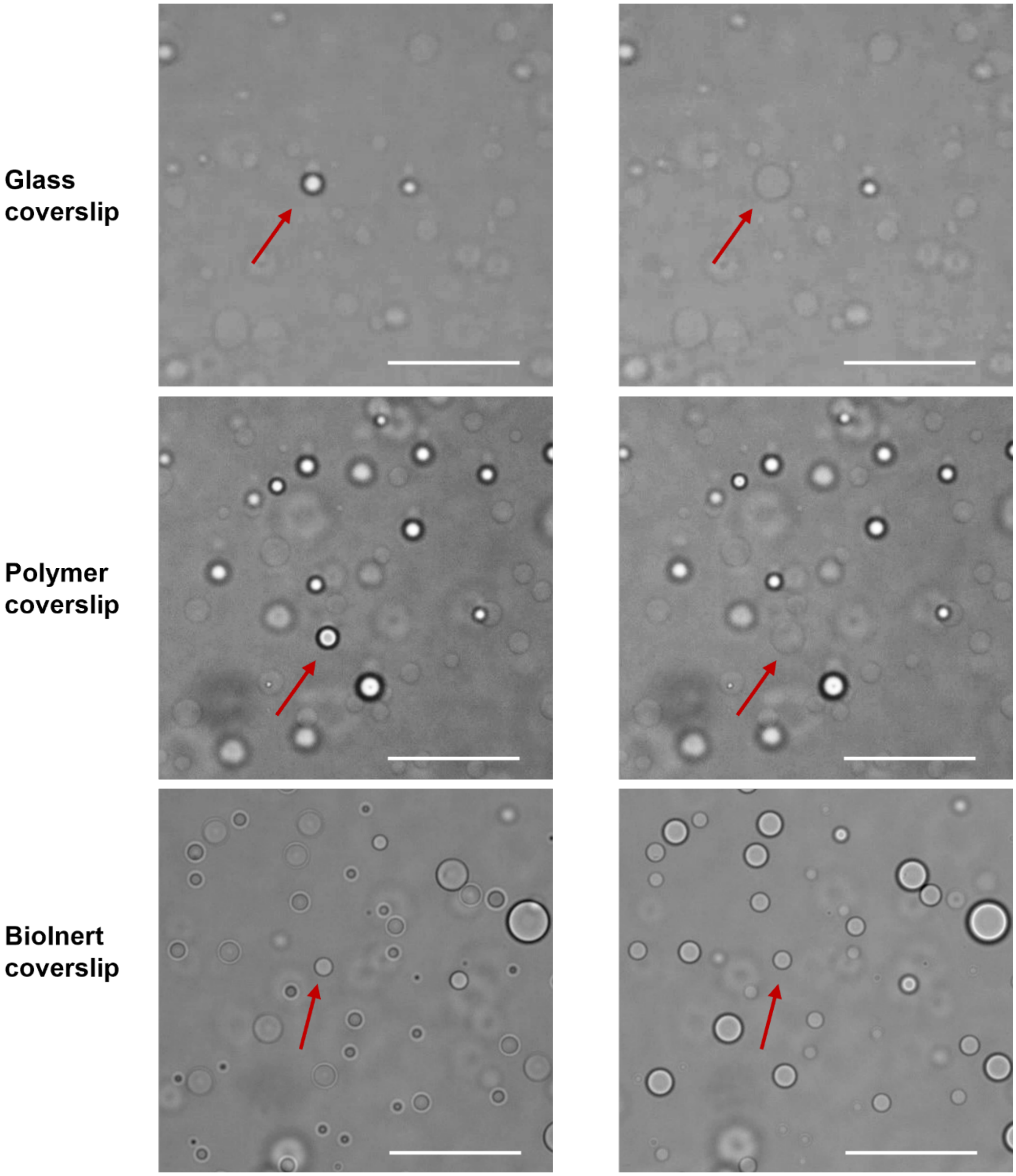

Supplementary Figure 30. Perturbation of condensate-surface interactions decreases the wettability of α-syn condensates. Representative DIC images of 60 μM FL α-syn with 25 μM PLK in phase separation buffer incubated at 37 °C in 8-well slides different coverslips. Images were acquired at the bottom surface of the well before (left) and after (right) a condensate in solution (indicated by a red arrow) wet the well surface. Scale bars represent 25 μm.

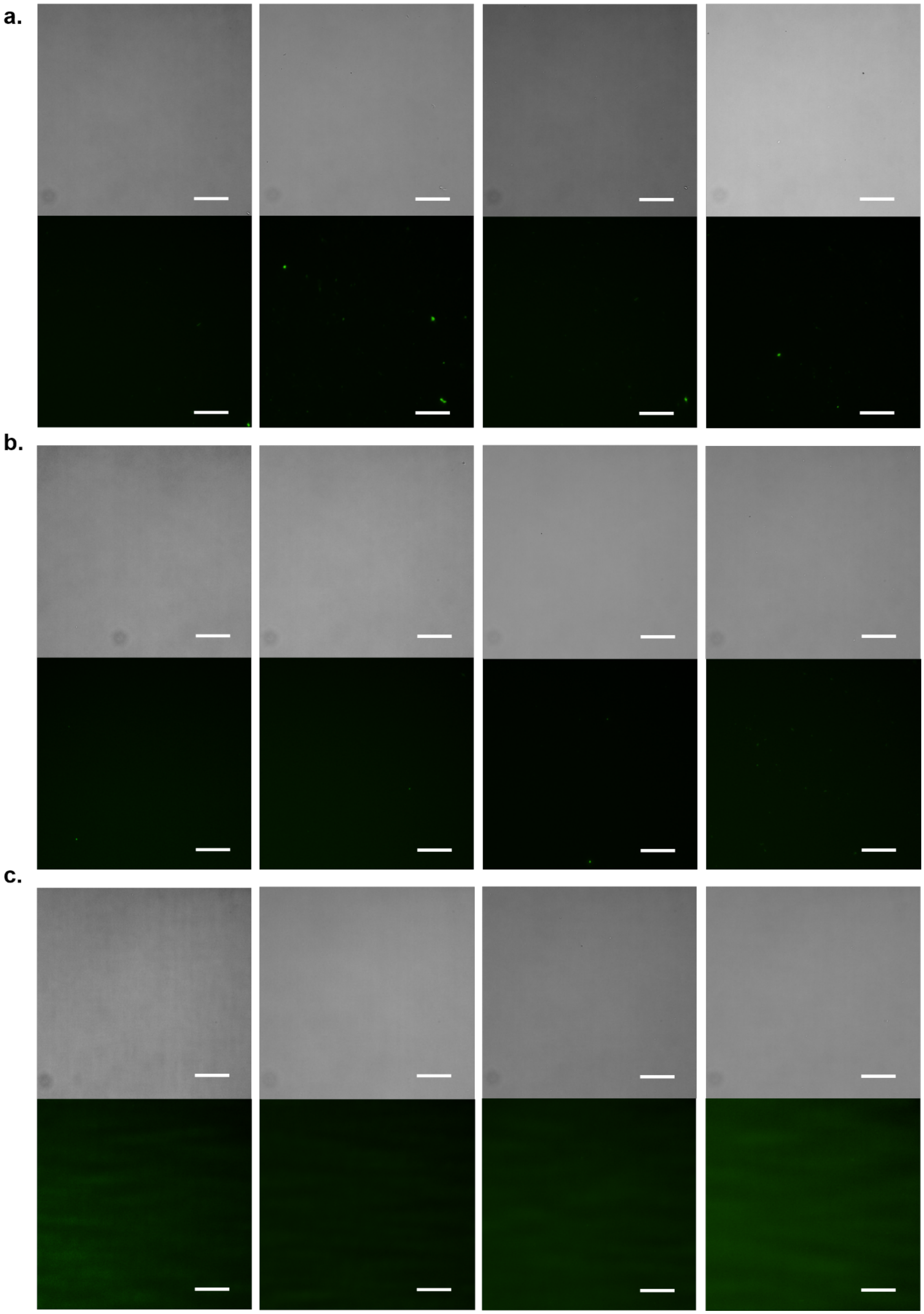

Supplementary Figure 31. Control samples for phase separation in the presence of different surfaces.

(a-c) Representative DIC (top) and fluorescent (bottom) images of phase separation buffer incubated at 37 °C in 8-well slides with a glass (a), polymer (b) or BioInert (c) coverslip. Images were acquired at selected time points at the bottom surface of the well. Scale bars represent 25 μm.

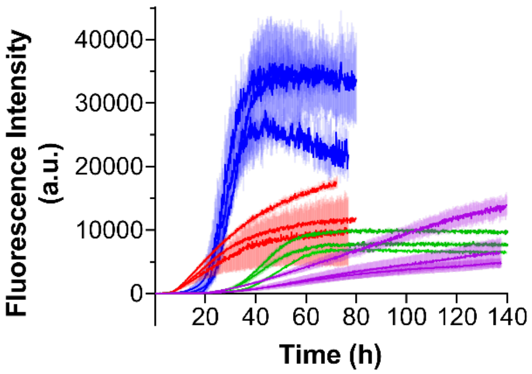

Supplementary Figure 32. N-terminal truncation modulates α-syn aggregation.

Raw dispersed solution amyloid aggregation of FL (blue), 5-140 (red), 11-140 (green) and 19-140 (purple) α-syn, monitored by ThT fluorescence intensity at 37 °C with agitation. Three individual biological repeats are shown per variant. Each repeat is the mean of either two or three technical replicates and semi-transparent error bars represent the standard deviation of the mean.

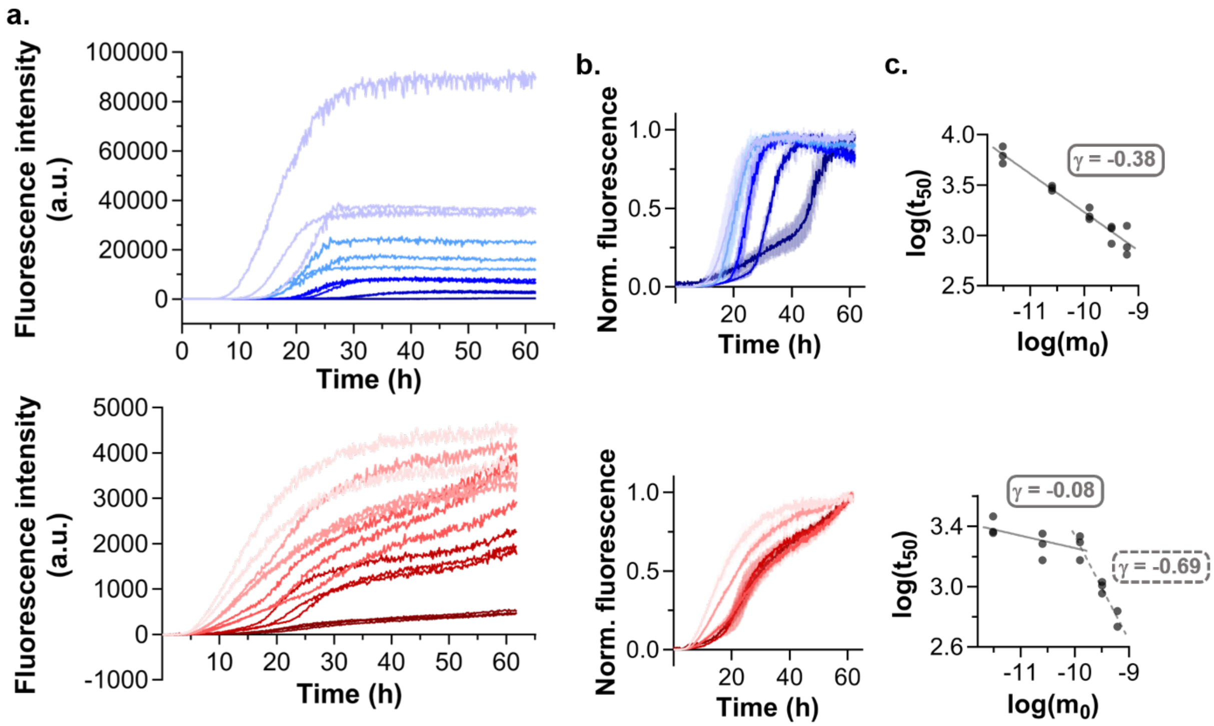

Supplementary Figure 33. 5-140 α-syn aggregation has an increased secondary nucleation contribution.

a, Raw dispersed solution amyloid aggregation data for varied concentrations (10, 25, 50, 75 and 100 μM) of FL (top, dark blue to light blue) and 5-140 (bottom, dark red to light red) α-syn, monitored by ThT fluorescence intensity at 37 °C with agitation. n ≥ 2 technical replicates from a single assay are shown. b, Normalization of the data shown in (a) for FL (top) and 5-140 (bottom) α-syn, where each curve is the mean of the technical replicates and semi-transparent error bars represent the standard deviation, n ≥ 2. c, Plot of the log of initial monomer concentration (m0) versus the log of the half-life of aggregation (t50), used to estimate the scaling exponent (γ) and provide insight into the mechanism of aggregation. n ≥ 2 technical replicates from a single assay are shown, corresponding to FL (top) and 5-140 (bottom) α-syn.

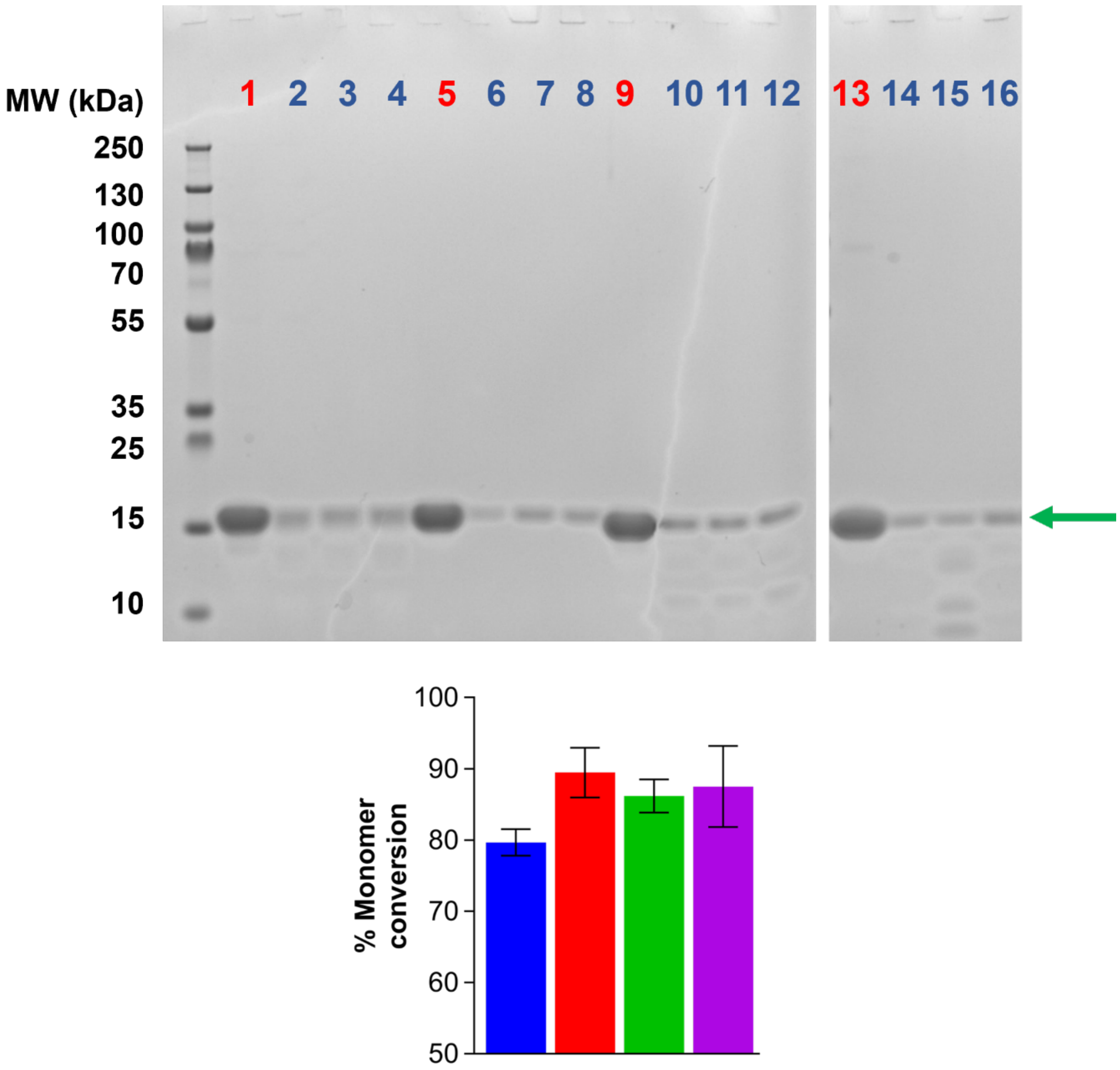

Supplementary Figure 34. N-terminal truncation does not affect α-syn monomer conversion.

Top: Representative SDS PAGE image of the soluble fraction of FL (lane 1-4), 5-140 (lane 5-8), 11-140 (lanes 9-12) and 19-140 (lanes 13-16) α-syn at the start (red) and end (blue) of a dispersed solution aggregation assay. Green arrow indicates the band quantified. The band intensity, background corrected using a blank area of gel, was measured using Fiji to estimate % conversion of α-syn monomer to insoluble aggregate. Bottom: corresponding quantification of the percentage conversion of soluble FL (blue), 5-140 (red), 11-140 (green) and 19-140 (purple) α-syn into insoluble aggregates during a dispersed solution aggregation assay. Representative biological repeat is shown. Each repeat is the mean of three technical replicates and error bars represent the standard deviation of the mean.

a.

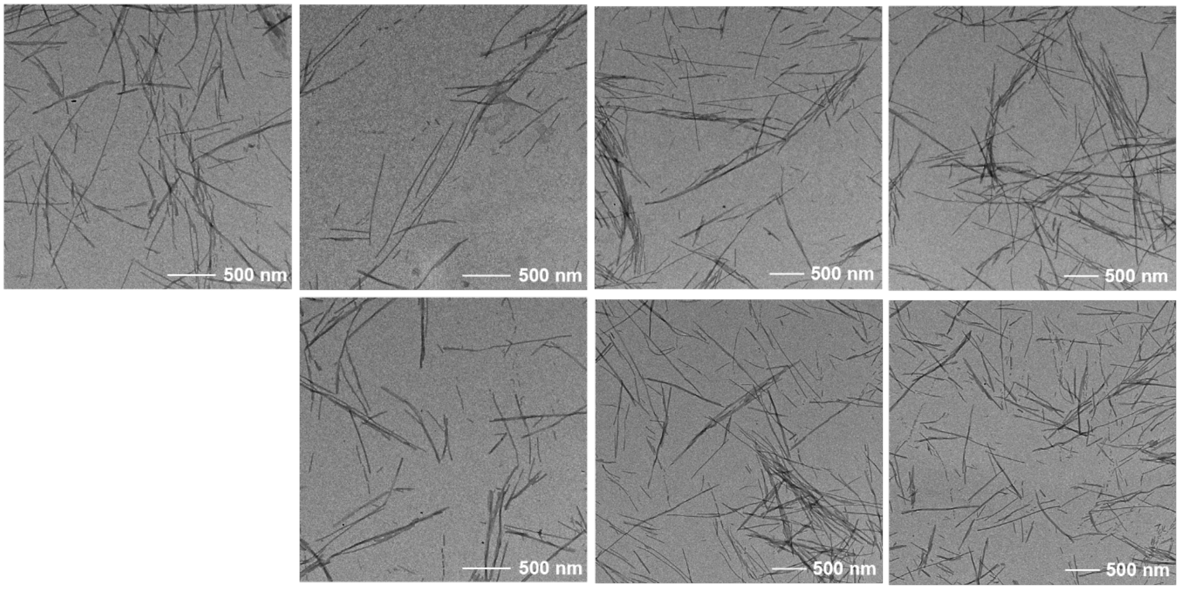

b.

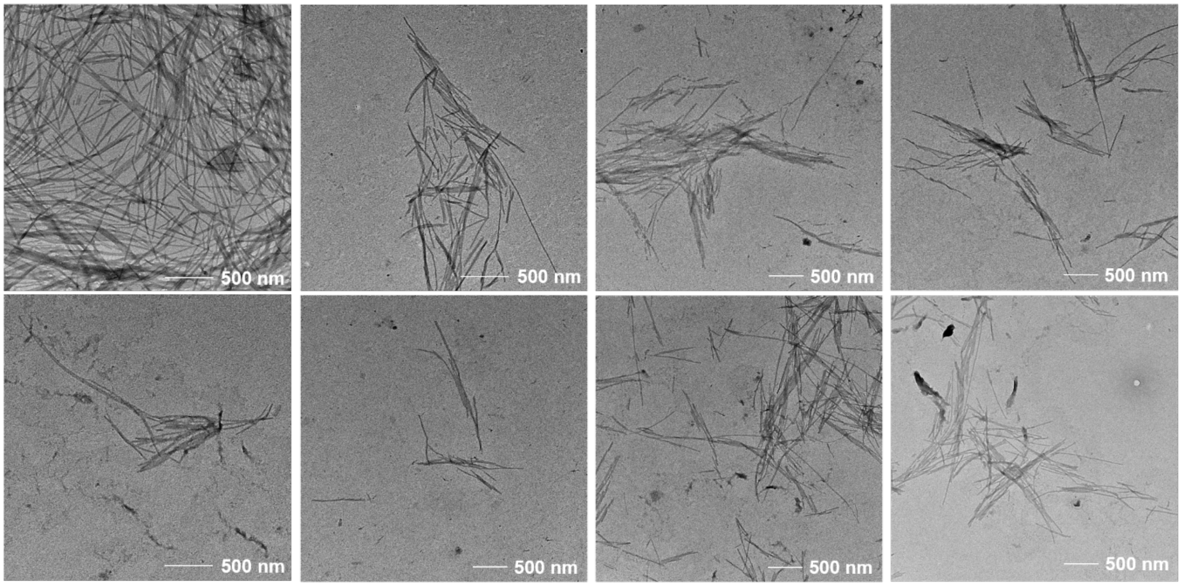

c.

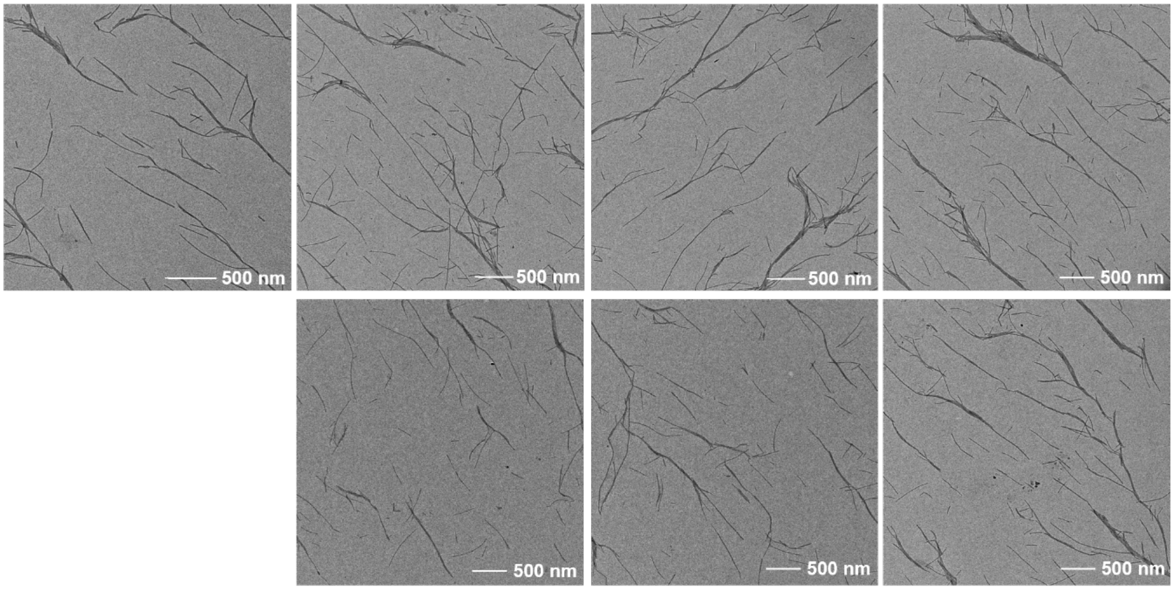

d.

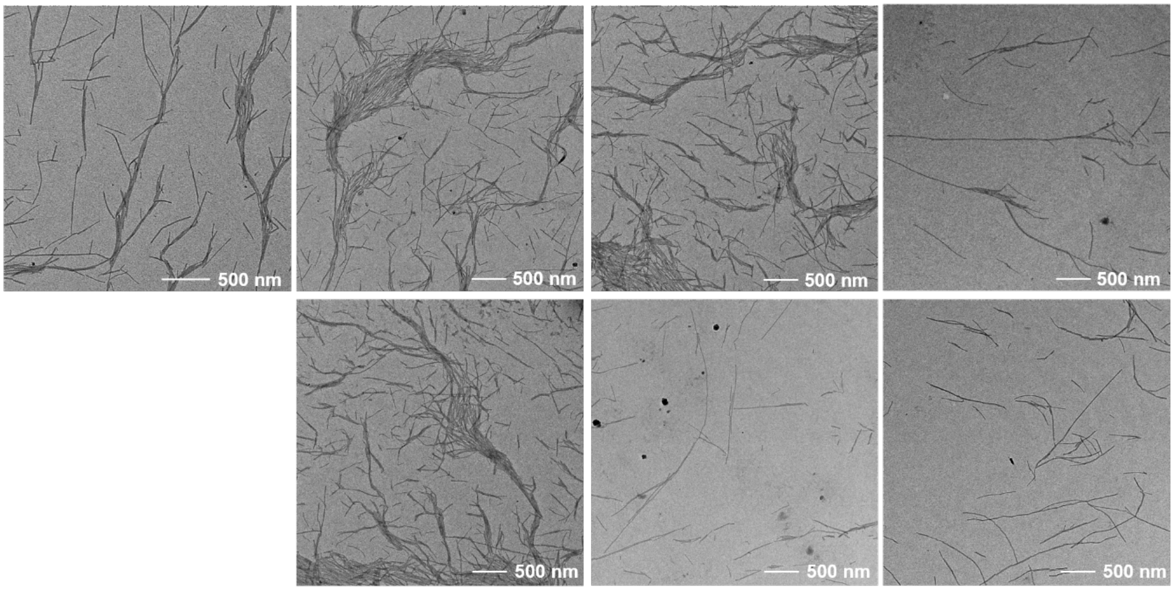

Supplementary Figure 35. N-terminal truncation affects α-syn amyloid morphology.

TEM images of FL (a), 5-140 (b), 11-40 (c) and 19-140 (d) α-syn taken at the end point of the dispersed solution aggregation assay and used for quantitative analysis of fibril length and width by Fiji. Scale bars represent 500 nm.

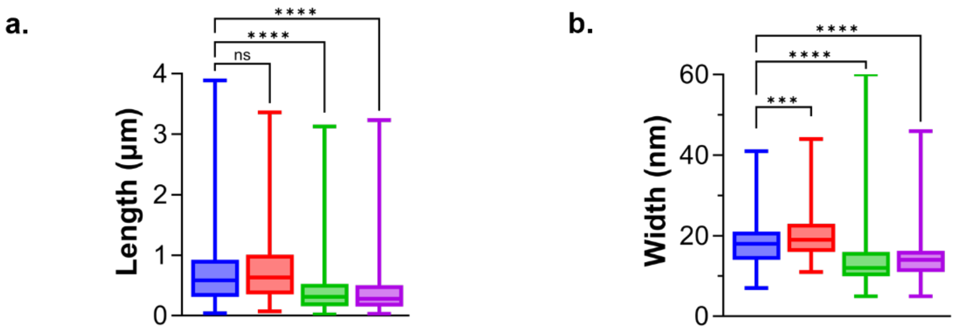

Supplementary Figure 36. Increased N-terminal truncation decreases the length and width of α-syn amyloids.

Length (a) and width (b) distributions of FL (blue), 5-140 (red), 11-140 (green) and 19-140 (purple) α-syn fibrils as determined by quantitative analysis performed on TEM images of the insoluble fraction at the endpoint of a dispersed solution aggregation assay (length: n = 419, 295, 476 and 524 respectively, width: n = 419, 292, 476 and 514 respectively). All TEM images quantified are shown in Figure 5 or Supplementary Figure 35. The box extends from the 25th to the 75th percentiles and the median is represented as a line. Error bars represent the minimum and maximum values.

FL and 5-140 α-syn fibrils are similar in length (median lengths 0.59 and 0.63 μm respectively), whereas 11-140 and 19-140 α-syn aggregates are typically significantly shorter (median lengths 0.31 and 0.28 μm, respectively). Although 5-140 and FL α-syn display similar median fibril widths, 18 and 19 nm, respectively, the distribution of FL α-syn fibril widths is significantly thinner than 5-140 α-syn. In contrast, 11-140 and 19-140 α-syn fibrils are thinner (median widths 12 and 14 nm, respectively).

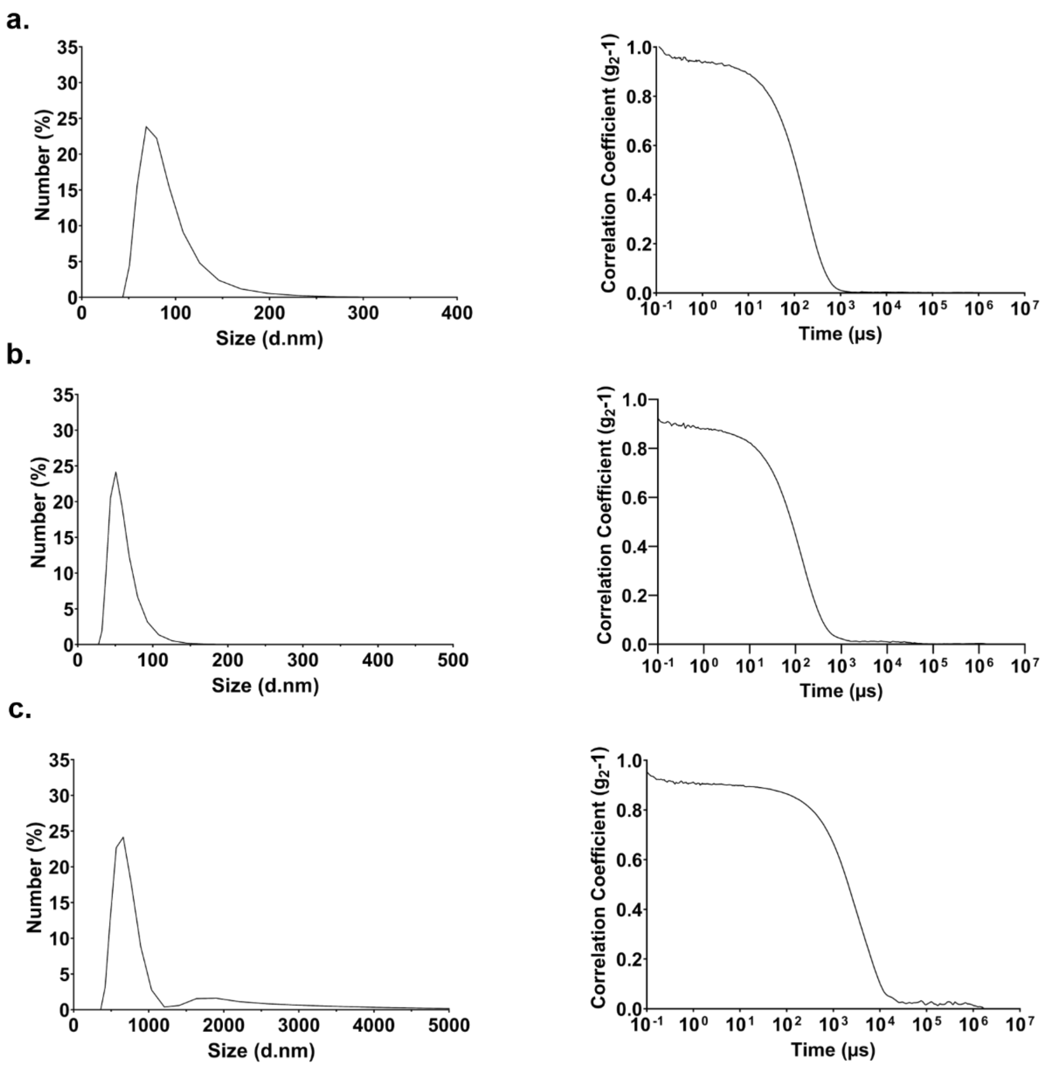

Supplementary Figure 37. Lipid vesicle and pre-formed fibril size confirmed by DLS.

Representative DLS data of (a) the DMPS lipid vesicles used to study primary nucleation, (b) short FL α-syn fibrils used for fibril elongation, or (c) long FL α-syn fibrils used for secondary nucleation. Number distribution is shown on the left and the corresponding correlogram on the right.

Supplementary References

1. R. J. Thrush, et al., A Facile Method to Produce N-Terminally Truncated α-Synuclein, *Front. Neurosci.*, 2022, **16**, 881480.
2. K. E. Paleologou, et al., Phosphorylation at Ser-129 but not the phosphomimics S129E/D inhibits the fibrillation of α-synuclein, *J. Biol. Chem.*, 2008, **283**, 16895-16905.
3. P. Mesquida, et al., Morphology and mechanical stability of amyloid-like peptide fibrils, *J. Mater. Sci. Mater. Med.*, 2007, **18**, 1325-1331.
4. G. Maurstad, et al., Dehydration stability of amyloid fibrils studied by AFM, *Eur. Biophys. J.*, 2009, **38**, 1135-1140.
5. N. J. Greenfield, Using circular dichroism spectra to estimate protein secondary structure, *Nat. Protoc.*, 2006, **1**, 2876-2890.
6. G. Meisl, et al., Molecular mechanisms of protein aggregation from global fitting of kinetic models, *Nat. Protoc.*, 2016, **11**, 252-272.
7. C. Galvagnion, et al., Chemical properties of lipids strongly affect the kinetics of the membrane-induced aggregation of α-synuclein, *Proc. Natl. Acad. Sci.*, 2016, **113**, 7065-7070.
8. P. Flagmeier, et al., Mutations associated with familial Parkinson’s disease alter the initiation and amplification steps of α-synuclein aggregation, *Proc. Natl. Acad. Sci.*, 2016, **113**, 10328-10333.
9. C. P. Doherty, et al., A short motif in the N-terminal region of α-synuclein is critical for both aggregation and function, *Nat. Struct. Mol. Biol.*, 2020, **27**, 249-259.
10. A. K. Buell, et al., Solution conditions determine the relative importance of nucleation and growth processes in α-synuclein aggregation, *Proc. Natl. Acad. Sci.*, 2014, **111**, 7671-7676.
11. J. Schindelin, et al., Fiji: an open-source platform for biological-image analysis, *Nat. Methods*, 2012, **9**, 676-682.
12. S. Ray, et al., Mass photometric detection and quantification of nanoscale α-synuclein phase separation, *Nat. Chem.*, 2023, **15**, 1306-1316.
13. P. Gonzalez-Tello, et al., Density and viscosity of concentrated aqueous solutions of polyethylene glycol, *Journal of Chemical and Engineering Data*, 1994, **39**, 611-614.
14. A. Eliassi, et al., Densities of poly (ethylene glycol)+ water mixtures in the 298.15− 328.15 K temperature range, *J. Chem. Eng. Data*, 1998, **43**, 719-721.
