# Supplementary material for: Surface wetting is a key determinant of α-synuclein condensate maturation
Source: Commun Chem. 2025 Nov 27;8:379. doi: 10.1038/s42004-025-01764-z (PMC12661020; doi:10.1038/s42004-025-01764-z)
Supplement: Supplementary file 3 — Description of Additional Supplementary Files [file 42004_2025_1764_MOESM3_ESM.pdf]

## Description of Additional Supplementary Files

File name- Supplementary Movie 1

File description – FL α-syn aggregate formation after phase separation. z-stack movie taken at the endpoint of a ThT assay under phase separation conditions when 60 μM FL α-syn was incubated with 25 μM PLK in LLPS buffer at 37 °C. The movie is an overlay of both DIC and ThT fluorescence channels and shows the formation of a large fibril network that extends across the bottom of the well and into solution. 2.5 μm z step, 64 μm z range, scale bar represents 50 μm.

File name- Supplementary Movie 2

File description – 2. 5-140 α-syn aggregate formation after phase separation. z-stack movie taken at the endpoint of a ThT assay under phase separation conditions when 60 μM 5-140 α-syn was incubated with 25 μM PLK in LLPS buffer at 37 °C. The movie is an overlay of both DIC and ThT fluorescence channels and shows the formation of a large fibril network that extends across the bottom of the well and into solution. 2.5 μm z step, 64 μm z range, scale bar represents 50 μm.

File name- Supplementary Movie 3

File description – 11-140 α-syn aggregate formation after phase separation. z-stack movie taken at the endpoint of a ThT assay under phase separation conditions when 60 μM 11-140 α-syn was incubated with 25 μM PLK in LLPS buffer at 37 °C. The movie is an overlay of both DIC and ThT fluorescence channels and shows the formation of a large fibril network that extends across the bottom of the well and into solution. 2.5 μm z step, 64 μm z range, scale bar represents 50 μm

File name- Supplementary Movie 4

File description – 19-140 α-syn aggregate formation after phase separation. z-stack movie taken at the endpoint of a ThT assay under phase separation conditions when 60 μM 19-140 α-syn was incubated with 25 μM PLK in LLPS

buffer at 37 °C. The movie is an overlay of both DIC and ThT fluorescence channels and shows the formation of a large fibril network that extends across the bottom of the well and into solution. 2.5 μm z step, 64 μm z range, scale bar represents 50 μm.
